# Fluidic torque–enabled object manipulation by microrobot collectives

**DOI:** 10.1126/sciadv.aea9947

**Published:** 2026-02-25

**Authors:** Steven Ceron, Gaurav Gardi, Kirstin Petersen, Metin Sitti

**Affiliations:** ^1^ Robotics Department, University of Michigan, Ann Arbor, MI 48109, USA.; ^2^ Physical Intelligence Department, Max Planck Institute for Intelligent Systems, 70569 Stuttgart, Germany.; ^3^ School of Electrical and Computer Engineering, Cornell University, Ithaca, NY 14853, USA.; ^4^ School of Medicine and College of Engineering, Koç University, 34450 Istanbul, Turkey.

## Abstract

Microscale systems experience strong viscous interactions because of the low–Reynolds-number regime in which they exist. This means that fluidic manipulation and actuation of passive objects can be enabled and influenced by the individual spin rate of microscale robots, the number of microrobots, and their positions relative to the objects. We explore these parameter spaces and find that the fluidic torque generated by a magnetic microrobot collective can be exploited to apply bidirectional torque to concentric ring structures and demonstrate this through physical experiments and numerical simulations. Additionally, we demonstrate how the fluidic torque of the microrobots can be exploited to actuate gear trains, rotate comparatively large three-dimensional objects, dynamically self-assemble internally driven ring structures, and absorb and expel large numbers of circular objects. Last, we show emergent behaviors where the microrobot collective’s morphology and method of locomotion changes as a function of the spin rate of the microrobots and the size and shape of the surrounding objects.

## Teaser

Spinning microrobot swarms harness fluidic torque for contactless motion, self-assembly, and advanced object manipulation.

## INTRODUCTION

Most microscale systems operate inside a fluid medium in the low–Reynolds-number regime and use physical contact to manipulate single passive objects (*1*–*4*). However, given the dominance of viscous effects over inertial effects in the low–Reynolds-number regime (*5*), it is advantageous to actuate passive objects through fluidic flows. Flow-based actuation can also enable the control of multiple passive objects at a time and induce interactions among them to enable dynamic self-assembly (*6*, *7*). Various mechanisms have been exploited to generate and manipulate fluid flows at small scales for microrobots (*8*, *9*), including acoustics (*10*, *11*), optics (*12*, *13*), magnetics (*14*–*16*), electro-hydrodynamics (*17*), self-propelling chemical micromotors (*18*), and electronically programmable elements (*19*). Mobile systems have been composed of single microrobots or collectives of microrobots that use flows to trap a single object and transport them (*20*–*23*). While useful, the inherent small size of microrobots prevents the transport of many objects or larger objects by a single robot. These limitations can be addressed by using a collective system composed of many robots that can each generate flow. Such collective systems have been shown to manipulate single objects larger than the collective’s constituents (*24*–*28*); however, despite their success in object rotation and translation, little has been done to explore how their actuation and manipulation capability scales with properties like collective size, spin rate, and shape relative to the objects that they are manipulating.

Here, we use a magnetic microrobot collective to explore these dependencies in practical experiments and through simulation, emphasizing their use in noncontact manipulation that may be helpful in delicate, small-scale object manipulation. Our collective consists of magnetic microdisks and has, in the past, been shown to reconfigure between various emergent behaviors, exploit heterogeneity in disk characteristics, and manipulate single passive objects (*25*, *26*, *29*, *30*). Previous studies have shown that magnetically actuated microrobot collectives can generate flow fields that can rotate nearby structures (*25*) and that microrobot size heterogeneity can drive transitions between particle expulsion and encapsulation (*30*); however, these prior works remained largely qualitative and did not quantify how collective-generated flows translate into measurable torque or systematic object manipulation. Here, we characterize and exploit the fluidic torque generated by rotating microrobot collectives to actuate and manipulate a range of passive objects with variable sizes and shapes and examine the objects’ responses when the microrobots are placed in various positions with respect to the object. We systematically explore the parameter space consisting of the rate at which the microrobots spin, whether they are inside, outside, or in between objects, and the number of microrobots in the collective. We find that fluidic torque can be exploited within concentric ring structures enabling them to exhibit co- and counterrotation, actuate gear systems and comparatively massive three-dimensional (3D) objects, dynamically self-assemble nonconcentric ring structures, and demonstrate how a dense, 1000-microrobot collective can be used to expel and aggregate or absorb and disperse many objects. We also simulate the concentric ring experiments and find that their behavior can be explained by low–Reynolds-number flows and that the rings’ rotation speeds are linearly proportional to the field frequency and the number of microrobots driving the system. Moreover, we find two behavior modes, dispersed rotation and aggregated crawling motion about an object perimeter, which depend on the shape and size of the object. The behaviors exhibited are possible due to the interaction between the externally driven microrobots and the fluidically driven structures, and the induced interactions among the structures. To reveal how fluidic torque generated by a microrobot collective governs multibody actuation and emergent motion, we adopt a hierarchical approach in which we first quantify the torque generated by a confined collective, then explore its transfer between multiple interacting structures, next demonstrate its use for functional manipulation of single or multiple structures, and lastly show how torque coupling with boundaries transforms the collective’s own behavior and morphology. Our results highlight a range of functionalities that can arise from exploiting fluidic torque generated by microrobot collectives for object manipulation, useful for packaging (*31*), assembly, or the simultaneous transport of many small-scale objects.

## RESULTS

### System overview

**Fig. 1.**
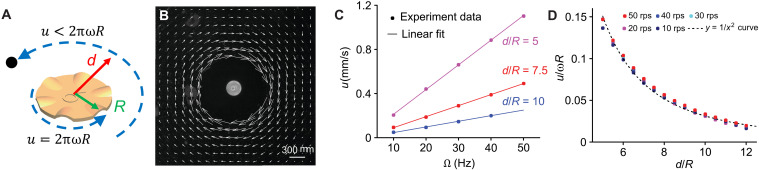
Flow visualization of a spinning single microdisk. (**A**) Representative graphic of a rotating microrobot and the rotating flows that it generates when spinning (with ω representing rotations per second) under a field frequency Ω. The fluid along the microrobot’s boundary (radius *R*) has a velocity approximately equal to 2πω*R* but drops as a power law with distance (1/*d*^2^) such that a tracer particle at some distance *d* will move with the fluid and have a velocity much lower than 2πω*R*. (**B**) Flow visualization using 3-μm diameter tracer particles around one spinning microrobot. Arrows represent the local flow speed and direction. (**C**) Flow velocity (*u*) as a function of magnetic field frequency for various distances away from the center of the microrobot (*d* is distance away from the microrobot’s center; *R* is the microrobot’s radius). (**D**) Flow velocity normalized by its value at the microrobot boundary (*u*/ω*R*) as a function of normalized distance away from the microrobot’s center for various field frequencies. Therefore, the flow velocity $u\propto \frac{2\pi \omega R}{{\left(\frac{d}{R}\right)}^{2}}$ as expected for a low–Reynolds-number flow (*35*). Note that the flow adjacent to the microrobot’s boundary is not included in (C) and (D) because it is too fast to be visualized using the current imaging hardware; however, the speed is estimated to follow the trends shown in these plots; at the boundary, it should match the speed of the rotating microrobot. rps, rotations per second.

Our microrobot collectives are made up of 300-μm-diameter magnetic microdisks at the air-water interface. These disks exert orientation-dependent magnetic dipole-dipole and capillary forces that are respectively attractive and repulsive on average and isotropic repulsive hydrodynamic forces on their neighbors. A rotating magnetic field vector enables each microrobot to spin about its center axis and create a circular flow field that imparts fluidic drag on its surroundings, influencing all other nearby microrobots and objects (Fig. 1A). Additional information about the system can be found in the “System overview” section in the Supplementary Materials. Figure 1B shows an experimental visualization of the flow field around a spinning microrobot. The flow speed (*u*) is proportional to the spinning speed (ω) of the microrobot (Fig. 1C), and it is inversely proportional to the square of the distance from the microrobot’s center (Fig. 1D). Therefore, the quick drop-off in the surrounding fluid’s velocity as a function of distance from the microrobot’s center limits a single microrobot from doing meaningful work on objects much larger than its diameter. By increasing the number of microrobots and positioning them inside or outside of large objects, we can create cumulative flow fields, which create enough drag to drive the rotational and translational motion of larger objects.

### Quantifying effects of fluidic torque through rotation of concentric structures

Our magnetic microrobot collective’s behavior under a rotating field in open space is well understood; past work (*29*) has shown that the microrobots organize themselves into a rotating group in which the neighbor spacing and the collective rotation correlates with the magnetic field’s rotation frequency. Here, we explore what happens when a rotating collective is placed in the presence of freely floating structures that are larger or smaller than the group’s coverage area and when the microrobots are placed inside or outside of freely floating rings. In these environments, the microrobots transfer hydrodynamic torque through azimuthal flow fields and cause the structure to rotate. The microrobots interact with each other and the surrounding structures in the low–Reynolds-number regime; therefore, if a microrobot collective is placed inside of a single ring structure, then the ring will rotate with the microrobots because the generated rotational fluidic torque is in the same direction. If a microrobot collective is placed outside of the structure, then the ring rotates opposite to the microrobots because the torque is reversed. For example, if the collective surrounds the ring structure and each microrobot is rotating clockwise, then the local flow fields are in the clockwise direction, which together create a global counterclockwise flow on the outside boundary of the structure and drive the structure to rotate in the counterclockwise direction. This enables us to exploit the flow behavior about the inner and outer perimeters of concentric ring structures and program each ring’s angular velocity (ω_ring_) as a function of the number of microrobots in the center and annulus regions and the field frequency driving them.

**Fig. 2.**
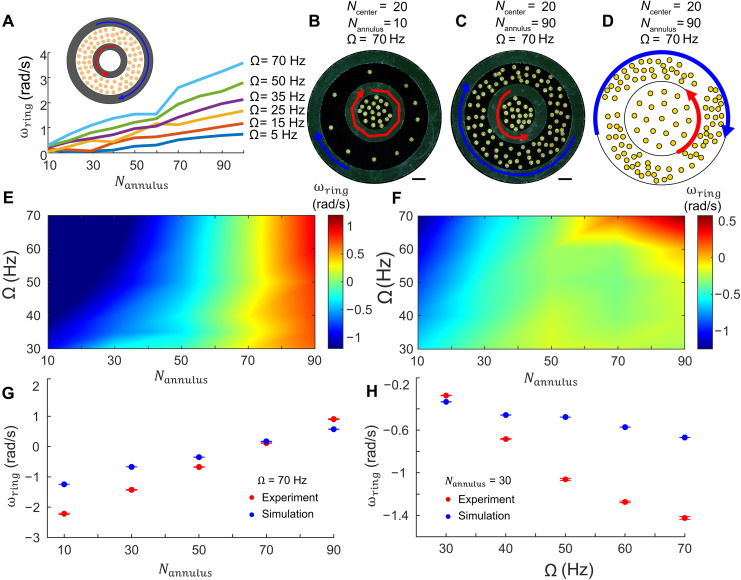
Co- and counterrotation of concentric rings by the microrobot collective. (**A**) Angular velocity (ω_ring_, units are rad per second) of the inner ring as a function of the number of microrobots in the annulus region (*N*_annulus_) when there are no microrobots in the center region (*N*_center_ = 0) for various field frequencies (Ω). (**B** and **C**) Experiment snapshots with various *N*_annulus_ and Ω when *N*_center_ = 20. (B) *N*_annulus_ = 10, Ω = 70 Hz. (C) *N*_annulus_ = 90, Ω = 70 Hz. In (B) and (C), the red and blue traces show the angular position over ~3.3 s of the inner and outer rings rotating, respectively; the scale bars correspond to 1 mm in length. (**D**) Simulation snapshot when *N*_center_ = 20, *N*_annulus_ = 90, and Ω = 70 Hz; the red and blue traces show the angular positions over 3 s. (**E**) Heatmap of the angular velocity (rad per second) of the inner ring in physical experiments when *N*_center_ = 20. (**F**) Heatmap of the angular velocity (rad per second) of the inner ring in simulations when *N*_center_ = 20. (**G**) Plots of angular velocity of the inner ring versus *N*_annulus_ when *N*_center_ = 20 and Ω = 70 Hz for experiments and simulations. (**H**) Plots of angular velocity of the inner ring versus Ω when *N*_center_ = 20 and *N*_annulus_ = 30 for experiments and simulations.

We show a representative set of key results from physical experiments and simulations in Fig. 2; detailed physical experiments and simulations with varying field frequencies (Ω), numbers of microrobots in the center (*N*_center_), and annulus (*N*_annulus_) regions and with up to three concentric ring structures are included in the Supplementary Materials (see figs. S2 to S14, movies S1 to S6, and the “Actuation of concentric ring structures” section). If *N*_annulus_ > 0 and *N*_center_ = 0, then the inner and outer rings rotate in opposite directions, with angular velocities proportional to *N*_annulus_ and Ω. As shown in Fig. 2A, the angular velocity of the inner ring is linearly correlated with *N*_annulus_ for a given field frequency. As the frequency increases, so does the slope of ω_ring_ versus *N*_annulus_ because the torque on each of the rings is linearly proportional to the field frequency that controls the flow speeds generated by each microrobot, which are shown in Fig. 1.

With two concentric rings and a field frequency of Ω = 70 Hz, the values of *N*_annulus_ and *N*_center_ determine both the direction and the magnitude of the angular velocity of the inner ring as shown in Fig. 2 (B and C). Here, the outer ring is influenced mainly by microrobots in the annulus region and rotates in the same direction as them, whereas the inner ring experiences competing torques from both the microrobots in the center and the annulus region. This configuration reveals nonlinear and nonintuitive hydrodynamic coupling between the rings, which corresponds to behavior that could not be anticipated from the superposition of the single-ring cases reported previously. When *N*_center_ = 20 and *N*_annulus_ = 10, the torque from the microrobots in the center dominates, and the inner ring rotates in the same direction as the outer ring (Fig. 2B), with the inner ring rotating much faster [~2 rad/s (115°/s faster)] than the outer ring. At *N*_annulus_ = 90, the torque from the microrobots in the annulus region dominates, and the inner ring rotates opposite the outer ring with a difference in rotational velocity of also ~2 rad/s (Fig. 2C). In addition to the angular velocity measurements presented here, we directly quantified the corresponding fluidic torque generated by the microrobot collectives. Across the parameter space explored, the measured torques ranged from ~10^–10^ to 10^–9^ N·m, with the largest torque occurring for Ω = 70 Hz and *N*_annulus_ = 90, where the outer ring experienced τ ~ 3.6 × 10^–9^ N·m. These values represent major amplification relative to the torque that would be imparted by a single microrobot and highlights the collective’s ability to generate substantial hydrodynamic work. Like the experimental results, a snapshot of the simulation with *N*_center_ = 20, *N*_annulus_ = 90, and Ω = 70 Hz demonstrates that the inner and outer rings rotate in opposite directions (Fig. 2D).

Because the torque generated by the microrobots is proportional to the field frequency, the difference in rotational velocities increases with higher frequencies as shown throughout the parameter sweeps in figs. S6 to S13, but the trends in behavior remain and are corroborated by our simulations as shown in Fig. 2 (E and F). For more details on the model and the simulation, results are included in the “Model for microrobots” and “Model for passive rings” sections in the Supplementary Materials, figs. S5 to S13, and movie S6. As shown in Fig. 2 (G and H), the simulations generally match our experimental trends well, although the angular velocity of the inner ring tends to be lower in simulation than observed in the physical experiments. The simulations predict the experimental results best at low *N*_annulus_; at high *N*_annulus_, the collective tends to break apart into several clusters around the inner ring’s circumference and apply torque about its perimeter. At lower Ω, the microrobots in the experiments tend to form localized clusters that do not span the circumference of the inner ring, and this is replicated well by the simulations. If *N*_annulus_ is low, however, then as Ω increases, the microrobots in the experiments tend to spread out and occupy the available annulus region, while the microrobots in the simulations tend to continue forming localized clusters that do not span the circumference of the inner ring; this decreases the torque on the simulated inner ring and minimizes the influence of the microrobots from the annulus region. The spreading in experiments is most likely because the rings affect the flow fields around the microrobots and cause them to spread out; however, that boundary effect is not explicitly known. This difference in the spreading of the collective at high Ω is likely the cause of the mismatch between the experiments and simulations.

The net flow generated at the surface of the rings depends on the positions and angular velocities of all the microrobots in the system; therefore, it is not trivial to know how these flows scale with the system size and whether this system still behaves within a low–Reynolds-number regime. The simulations demonstrate that the rotation speeds of the passive rings are linearly proportional to the number of microrobots in the annulus region when all other parameters are kept constant. This finding is supported by the experiments, and, although there is some quantitative disagreement, the simulations reproduce similar trends; this enabled us to highlight where the simulations disagree with experiments and which interaction terms are likely missing from the model. Because the simulations broadly capture the experimental trends, they provide qualitative insight into how the system parameters influence the rings’ rotation behaviors. Therefore, the simulations may serve as a useful guide for exploring regimes beyond those tested experimentally (e.g., faster rotating magnetic fields) and for narrowing down parameter ranges that are likely to produce specific behaviors such as co- or counterrotation. The agreement between the simulations and the experiments can be further improved by adding the boundary effect from the ring and by modeling the rings with finite thickness (instead of the infinitesimally thin rings in the current model) and remains as a topic for future research. Having established how flows generated by microrobot collectives produce torque that enables programmable ring rotational velocity and how that rotation velocity changes as a function of microrobot number and field frequency, we next explore how the same mechanism can be harnessed to drive and manipulate external structures.

### Functional exploitation of fluidic torque for multibody manipulation

**Fig. 3.**
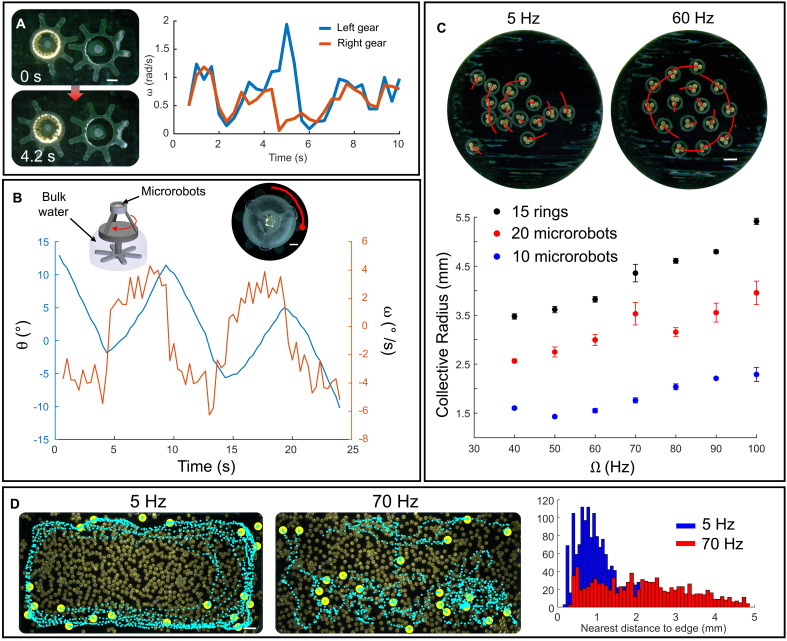
Actuation, self-assembly, and manipulation using the microrobot collective. (**A**) Fixed gear system in which a 40-microrobot collective is inside of the gear on the left with half of its teeth missing, and it drives the motion of the gear on the right. Right: Plot of instantaneous angular velocities of the left and right gears during a full rotation of the left gear. (**B**) Actuation of a 3D object. Top left: Graphic representation of a 3D object with a section of its body inside the bulk fluid and another section outside; a microrobot collective is placed in a small pool of fluid at the top of the object. Top right: Top view of the 3D object in experiments with 13 microrobots at the top of the object. Bottom: Angular orientation and angular speed over time of the 3D object. (**C**) Dynamic self-assembly. Top left: A cluster of ring structures rotating when Ω = 5 Hz. Top right: Ring structures in a circular rotating formation when Ω = 60 Hz. Bottom: Collective radius of the ring structures as a function of the field frequency compared to that of microrobot collectives. (**D**) Object manipulation with a 1000-microrobot collective. Left: Snapshot of a 1000 microrobot collective driven at 5 Hz enabling 20 objects to move toward the edges of the collective. Middle: The collective driven at 70 Hz and dispersing the 20 objects throughout its coverage area. Right: Histogram of passive particles’ nearest distance to arena edge when Ω = 5 and 70 Hz. All scale bars correspond to 1 mm in length.

Building directly on the experiments above, we demonstrate how fluidic torque can be functionally exploited for multibody actuation and object manipulation in increasingly complex geometries. Fluidic torque–enabled actuation of traditional mechanical elements is demonstrated in Fig. 3A; a 40-microrobot collective generates flows that rotate the gear in which it lies. Upon actuation, the gear turns another gear through a combination of physical contact and capillary interactions. The instantaneous angular velocity for the driven gear roughly doubles when its teeth are not in contact with the second gear and the load drops. The gears have fixed centers that enable them to rotate about their center point. Although similar rotational motion was observed in previous work (*25*), the mechanism here is fundamentally different. The earlier study involved two free-floating rings that interacted through capillary coupling at a single contact point; however, the present setup uses gear-like structures that each rotate about their own fixed axis and mechanically couple to their neighboring gear. This configuration thus functions as a controllable, contact-driven gearing system rather than a free-floating, capillary-driven interaction. As shown in the “Description of experiments with gear-like systems” section in the Supplementary Materials, fig. S15, and movie S7, it is possible to drive greater numbers of gears, a gripper, and actuate a pinion to move along a rack. Fluidic torque–enabled actuation can also be applied when the microrobot collectives are separated away from the bulk fluid, as shown in the 3D object in Fig. 3B, where 13 microrobots were placed within a small film of liquid atop a floating object. When driven at Ω = 70 Hz, the microrobots can rotate the object in alternating directions at up to ~4°/s, as shown in Fig. 3B. It is worth noting the object weighs more than 45,000 times that of a microrobot.

The use of fluidic torque–enabled actuation with microrobot collectives also permits easy customization and hierarchical design. Figure 3C shows examples where 15 free-floating rings are driven by three microrobots each to apply higher torque than a single-disk can. Because of the hierarchical setup, when the microrobots spin, each actuated ring, in turn, generates flows on the neighboring structures, which allows the whole group to dynamically self-assemble into organized rotating groups with a programmable collective radius and neighbor distance. At low field frequencies, the rings cluster, whereas, at higher frequencies, they rotate. The rings’ collective radius (Fig. 3C) and neighbor spacing trends mimic the trends of 10 and 20 free-floating microrobots because the motion induced by the microrobots within each ring causes the same type of flows to be generated on the ring’s exterior, but at a larger length scale. For more information on this behavior see the “Characterization of dynamic self-assembly experiments” section in the Supplementary Materials, figs. S16 and S17, and movie S8.

Last, we study how the hydrodynamic repulsion and boundary effects can be harnessed to absorb and expel passive objects from a collective by exploiting the fluidic torque generated by a dense 1000 microrobot collective intermixed with 20 1-mm-diameter objects. At low frequencies (Ω = 5 Hz), the microrobots rotate slowly, generating weak shear and, thus, low hydrodynamic repulsion, which enables the collective to remain compact and dominated by the microrobots’ pairwise attractive magnetic and capillary interactions, in turn expelling the larger objects toward the perimeter. At higher field frequencies (Ω = 70 Hz), faster rotation strengthens the flow fields and shear between neighboring microrobots, which increases the viscous stresses and lubrication pressure and enhances hydrodynamic repulsion that drives the microrobots to move away from each other. At low density, the higher repulsion would lead the collective to simply occupy a larger area, but, at high density, the confinement drives the microrobots to disperse in a disorganized fashion, in turn absorbing the objects.

It is worth noting that arena space constraints cause the azimuthal flow fields to be affected by the arena boundary, which causes the collective to split up into many small rotating clusters. As the microrobots spin close to each other at lower frequencies, their azimuthal flow fields combine on the perimeter which helps to pull the passive particles toward the exterior. When the field frequency increases, the multiple rotating clusters disorganize the azimuthal flow fields so that the passive particles spread throughout the entire arena. While this frequency-dependent transition between repulsion and attraction resembles the encapsulation/expulsion behavior observed in an earlier work with a single particle (*30*), the underlying mechanism here is fundamentally different. The previous work involved a sparse collective heterogeneous by size, and the observed expulsion and encapsulation behaviors arose from size-dependent hydrodynamic asymmetries. The encapsulation and expulsion behavior observed here, however, originates purely from changes in the collective’s generated flow field and fluidic torque. At low frequencies, the slowly rotating, spatially extended vortex produces outward shear that repels surrounding objects, while, at higher frequencies, stronger azimuthal flows and induced recirculation zones that pull the larger passive bodies toward the center. This mechanism, therefore, represents a collective-scale hydrodynamic phenomenon rather than a particle-scale interaction. Additional details are included in the “Characterization of object absorption and expulsion with a 1000-microrobot collective” section in the Supplementary Materials, figs. S18 to S26, and movies S9 to S11. The particle-boundary distance distributions in Fig. 3D and figs. S21 and S24 show this tendency. Here, the distance is measured between each passive particle and the nearest arena edge. As the field frequency increases, the distribution flattens out to resemble a uniform distribution, which means that the particles are spread throughout the arena and have many particles in the center and outer regions.

### Feedback between fluidic torque and collective morphology

**Fig. 4.**
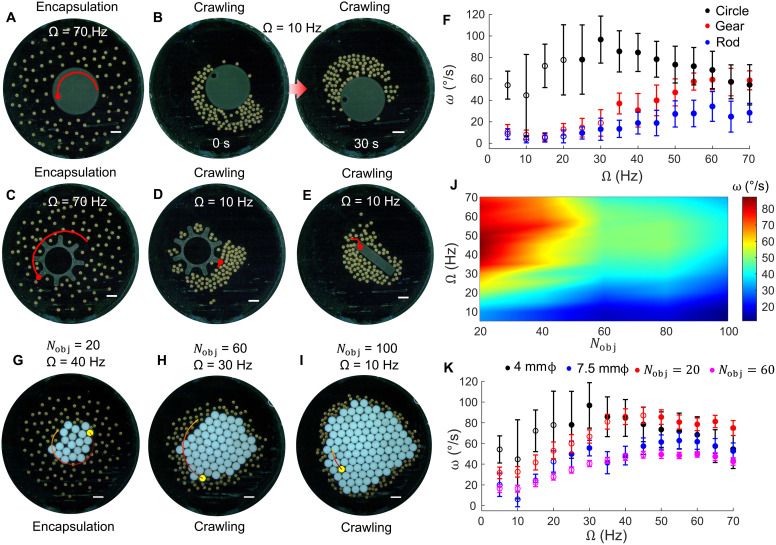
Microrobot collective morphology reconfiguration about object’s perimeter. (**A**) Encapsulation of a circular object (diameter of 4 mm, Ω = 70 Hz). (**B**) Clustering and crawling-like motion about a circular object’s perimeter (diameter of 4 mm, Ω = 10 Hz). (**C**) Encapsulation of a gear-shaped object (outer diameter of 4.3 mm, Ω = 70 Hz). Crawling about a (**D**) gear-shaped object and a (**E**) rod-shaped object (length of 4 mm). (**F**) Angular velocity of the circular, gear, and rod objects shown in (A) to (E) across many frequencies; the hollow circles are the instances where the microrobot collective is in a crawling state and the filled circles are when the collective is in a dispersed, encapsulating state. (**G** to **I**) Rotation of aggregating circular objects by a 100-microrobot collective during 10-s durations. (G) *N*_obj_ = 20, Ω = 40 Hz. (H) *N*_obj_ = 60, Ω = 30 Hz. (I) *N*_obj_ = 100, Ω = 10 Hz. (**J**) Heatmap of angular velocity in degrees per second across the *N*_obj_ − Ω parameter space corresponding to the experiments in (G) to (I). (**K**) Angular velocity of two circular objects with diameters of 4 and 7.5 mm, and aggregates of *N*_obj_ = 20 and *N*_obj_ = 60, which each have comparable areas to the circular objects, respectively. Data points with hollow circles correspond to frequencies at which the collective is in a crawling state, and filled circles correspond to when the collective is in an encapsulation state. All scale bars correspond to 1 mm in length.

Beyond driving passive structures, fluidic torque also couples back to the collective itself. Therefore, we next examine how interactions with boundaries and object shape affect the collective’s organization and motion. We present an emergent behavior, in which a collective alters its motion modality with respect to large, passive objects, from dispersed rotation about the object perimeter to an aggregated “crawling” motion (Fig. 4). We found that the previously unknown “crawling” mode of locomotion persisted in a 100-microrobot collective across small and large objects and is affected by the object’s corrugations.

At high field frequencies, the microrobots spin fast, and the hydrodynamic repulsion dominates their mutual interaction, which causes them to disperse and encapsulate the object (Fig. 4, A and C). Once the collective encapsulates the object, it exerts symmetric torque along the length of its perimeter and enables maximum object rotation. At low field frequencies, however, a crawling modality appears, stemming from the low hydrodynamic repulsion between microrobots, which leads to higher clustering where the attractive magnetic dipole-dipole and capillary interactions between the microrobots dominate and enable the collective to stick together along the edge of the object (Fig. 4, B, D, and E). Using this crawling mode, the collective can transport itself along the object to the other side of the arena while rotating the object minimally. The crawling mode observed here emerges because of the fluidic torque generated by the collective, symmetry breaking, and the microrobots’ capillary interaction with the object, and this enables the group to crawl about a curved, complex structure along its convex and concave features although it is free to rotate and translate.

Figure 4F shows how the collective’s ability to rotate an object depends on the object’s shape although the circle, gear, and rod object have a similar characteristic length of ~4 mm. The circular object has a much higher angular velocity than the gear and the rod, starting at the low frequencies, because the gear has concave regions that trap small clusters of microrobots and force uneven fluidic torque about the perimeter of the object, and the rod splits the collective into two groups of different sizes that enable the rod to translate rather than rotate. At higher frequencies, all objects tend to settle at approximately the same angular velocity because the microrobot collective has dispersed and encapsulated the object and, thus, exerts evenly distributed torque about the object’s perimeter.

When we systematically increase the area of a central object by adding circular objects that aggregate with each other and form a single mass, we observe that, when the aggregate is a smaller size, the collective can rotate it at a faster rate than bigger aggregates (Fig. 4, G to K). The frequency at which microrobots transition from a crawling state to an encapsulating state is higher for an aggregate than for a single circular object of the same size (Fig. 4K) because the combination of convex and concave regions at the aggregate’s boundary traps small clusters of microrobots. Additional details are included in the “Discussion on collective crawling about passive objects” section in the Supplementary Materials. The objects’ rotations and the microrobots’ crawling behavior are shown in movie S12.

Our experiments reveal a continuous progression from torque generation to torque exploitation and lastly to feedback between torque and passive structure geometry. The single- and concentric-ring experiments establish the quantitative relationship between microrobot number, frequency, and torque, while the gear and encapsulation demonstrations show how this torque can be harnessed for controlled multibody actuation, and the crawling-mode experiments highlight how torque coupling with boundaries can transform the collective’s own morphology. This hierarchy illustrates that fluidic torque can be a unifying mechanism underlying both object manipulation and emergent collective motion.

## DISCUSSION

The results presented in Figs. 2 to 4 follow a natural hierarchy, beginning with a quantification of the generated fluidic torque, progressing to its controlled transfer and use in multibody systems, and culminating in a form of collective motion. This unified framework establishes fluidic torque as both a measurable and a programmable mechanism for microrobot collectives. We exploited the fluidic torque generated by a rotating microrobot collective to actuate and manipulate multiple passive objects, including large 3D structures, in the surrounding environment. We demonstrated flow-driven, programmable co- and counterrotation of concentric ring structures. We replicate and explain the programmable rotation of the concentric ring structures through a physical model, and this helps us better understand the fluid-based interactions between the microrobots and the objects that enable the actuation, manipulation, and morphology reconfiguration capabilities. Our study also demonstrated that fluidic torque–based actuation can further cause contact-based and flow-based interactions between multiple passive objects. Last, we demonstrate that the microrobots’ fluidic interactions with objects of different geometry can alter the collective’s morphology.

While prior works (*32*–*34*) have used flows to trap or transport small particles, we quantify and exploit fluidic torque from a dense, homogeneous microrobot collective to actuate and rotate passive bodies larger than the individual microrobots and the collective, including concentric rings, gear-like structures, 3D structures, and circular structures. This establishes noncontact torque transfer, which is distinct from flow trapping, as a programmable mechanism for multibody actuation. The present work focuses mostly on how objects can be manipulated to exhibit rotational motion; however, translational motion can also be generated by enabling asymmetric flows about the perimeter of an object, e.g., by placing the microrobots on one side of an object. These asymmetries generate a net lateral fluidic force, which enable the object to translate along a desired direction at a specific speed, and quantifying this type of motion is an important direction for future studies. This study furthers our understanding of fluid-structure interactions at small scales and demonstrates how these interactions affect the passive objects present in flow fields of microrobot collectives. We quantify the structures’ motions through series of experiments so that, in future works, fluidic torque can be viewed as more than just a by-product of the microrobots’ rotational collective motion, but as a tunable mechanism for actuation and self-organization. Although our experiments were run at a fluid-air interface, the fluidic-torque mechanism is driven by bulk hydrodynamic interactions, and, thus, the same qualitative behaviors should emerge in submerged environments or liquid-liquid interfaces, with viscosity determining the quantitative scaling. Insights into the underlying interactions between microrobot collectives and their surroundings are critical to realizing micro- and milliscale part manipulation and assembly in small-scale desktop manufacturing, packaging, and biomedicine.

## MATERIALS AND METHODS

### Fluidic torque calculation

To estimate the rotational fluidic torque exerted on each passive ring, we used the classical low–Reynolds-number expression for the drag torque on a rotating microdisk

$$\tau =8\pi \eta R_{\text{ring}}^{3}\;\omega_{\text{ring}}$$

Here, η is the viscosity of water, $R_{\text{ring}}$ is the radius of the ring, and $\omega_{\text{ring}}$ is the ring’s experimentally measured angular velocity. Although this expression is derived for a sphere in ideal Stokes flow, it provides an appropriate approximation for our system because, although our Reynolds number is small but nonzero, the physical experiments and simulations demonstrate that the system operates in a regime dominated by viscous forces. The flow velocity around a single microrobot decays monotonically, and the rings’ angular velocities scale linearly with the driving field frequency if the number of microrobots is constant; this indicates that the system is demonstrating Stokesian behavior, and, thus, the inertial effects remain negligible. The exact values of the fluidic torque generated by the collective may differ depending on the true drag coefficients of the microrobots; however, the general magnitude and trends observed throughout the study will remain the same.

### Microrobot fabrication

The microrobots were fabricated by the Nanoscribe Photonic Professional GT 3D printer (two-photon polymerization system) and sputtered with nanofilms of 500-nm cobalt and 60-nm gold using a sputtering machine (Kurt J. Lesker NANO 36).

### Video acquisition and analysis

The experiments were performed under a Leica manual zoom microscope Z16 APO, and they were recorded using a Basler acA2500-60uc camera. A light-emitting diode light source SugarCUBE Ultra illuminator was connected to a ring light guide [0.83-inch (2.1082-cm) inside diameter, Edmund Optics #54-176] and was used to illuminate the microrobots. A Python script was developed using the OpenCV library to process the experimental videos and extract the positions of the microrobots. The positions of the passive objects were tracked using a MATLAB script. The analysis of the processed data was done using MATLAB, and the raw images were used without any enhancements for the processing. All the heatmaps and plots corresponding to experiments in the Results section and Supplementary Materials originate from discrete experimental measurements. Almost all experimental data related to the passive structures’ motions was processed by manually clicking on elements to determine the rotational and translational motion of the passive structures. The exception was the processing for the experiments corresponding to Fig. 4 (G to K) because the white passive particles’ motions could be reliably tracked through a MATLAB script. All experiment videos reported in the Supplementary Materials have a playback speed of 1×.

### Experimental protocol

For each frequency test, the microrobots were first driven at 70 Hz for about 20 s to disorganize the collective and then driven at the frequency of interest for about 20 s before capturing the collective behavior in a steady state. For the concentric ring rotation portion of the study, each experiment was recorded for 10 s at 30 frames per second. The orientation of each ring was manually tracked every 10 frames using a laser-cut circular marker on each ring, which gave 30 data points per experiment, and the angular velocity and fluidic torque were computed from this data. The heatmap in Fig. 2E was generated by interpolating between discrete experimental measurements using MATLAB’s interpolation function, which results in a continuous surface representation of angular velocity across the tested parameter space. The experiments involving 1000 microrobots were conducted in a rectangular arena, and the microrobots were first driven at 70 Hz for about 20 s for each frequency test to disorganize the collective and the passive objects; the microrobots were then driven at the frequency of interest for about 20 to 30 s before capturing the positions of the passive particles when the collective was at steady state. Each experiment was recorded for 10 s at 30 frames per second. The microrobots do not step out until the frequency is above 120 Hz, and all experiments are run with a frequency less than that value; therefore, all experiments occur below the step-out threshold, and we do not need to take the step-out dynamics into consideration.

Each 2D passive object that is gray was composed of 120-μm-thick polydimethylsiloxane (PDMS) with mixed carbon nanotubes to allow for better clarity in the video recording. The passive objects were laser cut using a LPKF U3 and then cleaned with 90% Isopropyl alcohol (IPA) to remove any impurities that could lead to unwanted capillary interactions with the microrobots. The 3D object was printed with VeroBlue material using the Formlabs printer. The fixed gears were fabricated by 3D printing an arena with 1-mm-diameter poles sticking straight upward and then inserting a PDMS ring with an outer diameter of ~2 mm over each pole so that it stayed at the same height as the water level. Each circular PDMS passive particle was laser cut to 800 μm. The white passive particles were made by mixing a white dye with the PDMS, which made them heavier than the other passive structures throughout the paper made with carbon nanotubes and, thus, enabled stronger capillary attractions that led to aggregation.
