# Supplementary material for: Fluidic torque–enabled object manipulation by microrobot collectives
Source: Sci Adv. 2026 Feb 25;12(9):eaea9947. doi: 10.1126/sciadv.aea9947 (PMC12935057; doi:10.1126/sciadv.aea9947)
Supplement: Supplementary file 1 — Supplementary Text Figs. S1 to S26 Legends for movies S1 to S12 [file sciadv.aea9947_sm.pdf]

Science Advances
AAAS

Supplementary Materials for

Fluidic torque–enabled object manipulation by microrobot collectives

Steven Ceron *et al.*

Corresponding author: Kirstin Petersen, kirstin@cornell.edu; Metin Sitti, sitti@is.mpg.de

Sci. Adv. **12**, eaea9947 (2026)
DOI: 10.1126/sciadv.aea9947

The PDF file includes:

Supplementary Text
Figs. S1 to S26
Legends for movies S1 to S12

Other Supplementary Material for this manuscript includes the following:

Movies S1 to S12

Supplementary Text

System Overview

We explore the effect and use of fluidic-torque in swarms of micro-disks, sputter-coated with ferromagnetic material, and floating on a water-air interface. The interactions between the microrobots are governed by the external magnetic field profile imposed on the collective by two pairs of perpendicular Helmholtz coils with adjustable magnetic field frequencies in the x and y directions (Ω_x and Ω_y). As shown in Eq. (1), the magnetic field vector (**B**) changes its orientation over time as a function of Ω_x and Ω_y; when $\Omega_x = \Omega_y = \Omega$, the magnetic field vector maintains a constant magnitude and rotates about the center of the workspace. In Eq. (1), B_x and B_y are the axial magnetic field amplitudes, which remain at 10 mT throughout this study, and t is time.

$$\mathbf{B}(t) = (B\cos(\Omega_\mathrm{x} t)) \cdot \hat{x} + (B\sin(\Omega_\mathrm{y} t)) \cdot \hat{y} \tag{1}$$

When $\Omega_x = \Omega_y$, the rotating magnetic field vector enables each microrobot to spin about its center axis; since the microrobots are on a fluid surface, they each create circular fluid flow profiles (or azimuthal flow fields) that influence all other microrobots and / or passive objects around them. When there are no passive objects present, the result is a circular collective rotating about its center. When there are passive objects in the vicinity, the collective affects the motions of the object (rotational and translational depending on its geometry) and the collective's motion is also affected by the fluid-structure interaction with the passive object.

Past work has explored the various collective behaviors that emerge across the Ω_x - Ω_y parameter space; some of these behaviors include chain-like structures, a globally static collective capable of encapsulating objects, and gas-like states for exploration of open areas. Here, we aim to explore and demonstrate the full benefits of fluid flows generated by the microrobots under a rotating magnetic field ($\Omega_x = \Omega_y$) to actuate and control single and multiple passive objects interacting through flow-based and contact-based interactions.

As shown through schematics in Fig. S1A, each microrobot generates a circulating flow field which exerts fluidic drag on surrounding robots and objects. The supplementary material contains additional results related to both the shape of the passive objects being manipulated and the morphology of the collective. Fig. S1B graphically represents how clockwise spinning behavior inside and outside an object enables counter-rotating flows, which generate fluidic torques in opposite directions which act on the object and affect its rotational motion. Fig. S1C graphically represents how a microrobot's spin can affect the rotational and translational motion of objects in the surrounding environment. Figs. S1D-H overview the various scenarios explored through this study which demonstrate the full extent to which microrobot collectives' rotational motion can be exploited to enable actuation, dynamic self-assembly, object manipulation, and collective morphology reconfiguration.

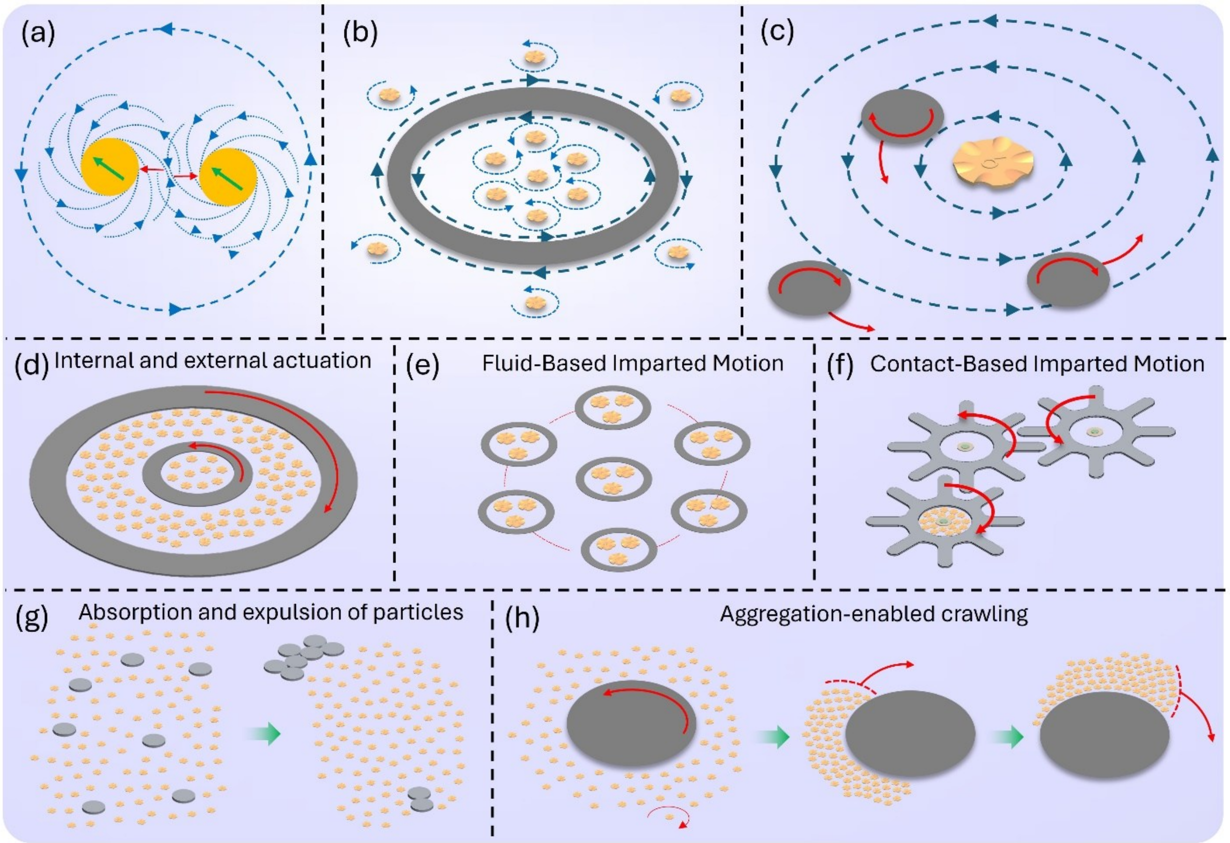

Fig. S1. Graphical overview of experimental realizations in this study. (a) Microrobots under a rotating magnetic field individually generate azimuthal flow fields which enables the collective to generate fluidic drag much more powerful than that generated by any single agent. **(b)** Rotating microrobots can enable rotational motion in passive objects and generate competing counter-rotating flow fields on the inside and outside of these objects. **(c)** Rotating microrobots can enable translational motion in passive objects through the circular flow fields. **(d)** Concentric structures can have programmable rotation depending on the density of microrobots within each of the regions and the microrobots' spin frequency. **(e)** Microrobots can drive multiple structures that use flow-based interactions to dynamically self-assemble. **(f)** Microrobot collectives can drive diverse small-scale structures with contact-based interactions. **(g)** Microrobot collectives can rotate, absorb, and disperse multiple passive objects or expel them to aggregate into clusters. **(h)** A microrobot collective can transition from crawling-like behavior along the surface of an object to encapsulating the object and rotating it through fluidic drag.

Actuation of Concentric Ring Structures

We experiment with torque transfer by placing varying numbers of micro-disks in passive, concentric rings. The microrobots transfer hydrodynamic torque through azimuthal flow fields and cause the concentric ring structures to rotate in clockwise or counterclockwise directions. The concentric structures are actuated within a circular arena that provides axisymmetric repulsion from the arena boundary and enables the structures to generally maintain a static center position close to the arena's center; this minimizes energy loss from object translation and maximizes object rotation. The following experimental results are shown with the corresponding simulation results through Figs. S2-S4 and Figs. S6-S14; Fig. S5 is a graphic representation of the physical model that enables us to reproduce the ring rotation behavior in the simulations.

When the microrobots are within the annulus region between two concentric ring structures, the collective cannot form a circular, rotating collective; instead, it can either form small clusters ($\Omega < 20$ Hz) or one continuous cluster about the circumference ($\Omega \geq 20$ Hz). At the lower frequency range, multiple clusters form because microrobots influence each other partially as a function of the passive object's shape. Rather than interacting with each other only in the world reference frame (e.g. the smallest distance between two microrobots), constituents largely influence each other around the perimeter of the passive structure, which causes the azimuthal flows from one microrobot to not influence other microrobots at a longer distance away than they would normally affect others in a structure-free environment since the fluidic torque will not transfer straight across through a passive object. When $\Omega < 20$ Hz, the hydrodynamic repulsion is low and prevents the microrobots from spreading out across the circumference of the ring structures. At higher frequencies, the microrobots exert much higher repulsion on each other, which causes the collective to spread out about the circumference. Moreover, the microrobots' higher spinning rate at higher frequencies increases the hydrodynamic drag from the ring's boundary which causes them to spread and encapsulate the ring.

When there is a high number of microrobots and a high frequency, it is intuitive that the encapsulation of the ring enables an evenly distributed hydrodynamic torque transfer about the ring structure's circumference; this enables continuous rotation with minimal translation. When there is a low number of microrobots and a low frequency, clusters tend to form unevenly about the circumference and thus ring structures move off center, exhibiting both rotational and translational motion because of the uneven distribution of fluidic drag. Therefore, it is worth noting through comprehensive experimental and simulation studies, that the purest rotational motion occurs when the density within the annulus regions is highest.

Our experiments dealing with the actuation of concentric ring structures illustrate three different types of cases: (1) when there are two ring structures and the microrobots are within the annulus region. (2) when there are two ring structures and the microrobots are within the center circular region and the annulus region, and (3) when there are three ring structures and the microrobots are within the center circular region and the two annulus regions. Throughout the experiments, and supported by simulations, we find that the direction and speed of rotation of each ring structure can be programmed by the density of microrobots within each region and the frequency; this means there are two parameters with which passive objects' behavior may be controlled.

Because of the local direction of the microrobots' rotating fluid flows, the outer ring receives torque that enables it to spin in the clockwise direction, while the inner ring experiences torque that enables it to spin counterclockwise. When microrobots are placed within the center region and the annulus regions; a ring's direction of rotation is dependent on the density of

microrobots in each of the concentric rings' regions. Except for the low frequency range, the experiments and simulations for test case (1) demonstrate that two ring structures always rotate in opposite directions because of the azimuthal flow fields within this area. The small, separate clusters that form at lower microrobot densities and cause the ring to rotate at a slower rate and the single, continuous cluster that forms at higher microrobot densities enables higher ring rotation speeds. The absolute difference in rotation speed between the inner and outer ring increases as a function of the number of microrobots within the annulus region and the magnetic field frequency.

The second test case is more complex; here, we find a three-way balance between the number of microrobots in the center region and annulus region, and the magnetic field frequency. When there is a high number of microrobots in the annulus region ($N_{annulus} = 90$) and a low number of microrobots in the center circular region ($N_{center} = 10$), the azimuthal flows from the microrobots in the annulus region driving the inner ring counterclockwise overcome the flows from the cluster in the center driving the ring in the clockwise direction. If we flip the experiment, and instead place a low density of microrobots in the annulus region ($N_{annulus} = 10$) and a high density in the center region ($N_{center} = 30$), the rings can be made to rotate in the same direction. If we increase the number of microrobots in the annulus region to $N_{annulus} = 50$, then the rings go back to rotating in opposite directions. Our comprehensive experimental study of the concentric rings' behavior includes a sweep of the frequency range from 10 to 70 Hz when there are 10, 20 and 30 microrobots in the center region. For each of these cases, we tested 10, 30, 50, 70, and 90 microrobots in the annulus region. At low $N_{annulus}$, the angular velocity of the inner ring is either zero or negative (inner ring spins in the clockwise direction) and becomes positive (inner ring spins in the counterclockwise direction) when $N_{annulus} > 30$; these fluctuations are because of the balance between the hydrodynamic torque exerted by the microrobots in the center region vs. the microrobots in the annulus region. The locations of the transitions shown in the heat maps are highly dependent on the inner and outer diameter of each of the rings since this dictates the boundary length along which the microrobots interact with the structure; however, the general trend is independent of the ring dimensions.

The third test case demonstrates several interesting behaviors that may arise from adding an extra concentric ring structure to the system, as shown in Fig. S14. The space constraints of the magnetic field's workspace increase the density of the microrobots within the concentric rings; this results in jamming behavior that can enable the rings to rotate in directions not consistent with the hydrodynamic torque the microrobots would normally exert. For example, we demonstrate that jamming can occur when there are 15 microrobots in the center region, 30 in the inner annulus region, and 60 in the outer annulus region. The trajectory in the figure only depicts the rotation of the rings during the last 1.7 seconds; however, the rotation of the inner ring changes midway through when four microrobots jam between the middle ring and the outer ring. The jamming is caused by similar mechanisms that lead to the crawling behavior observed in the latter experiments in the main text; the small spacing between the middle and outer ring disrupts the azimuthal flow fields and prohibits the microrobots from spreading out as much as they would in open space. Jamming happens because at low rotation speeds (and at very high local densities) the microrobots assemble and form a solid-like cluster and this cluster acts as a rigid, physical link between the inner and the outer rings that couples the motion of the two rings. The tight spacing forces them to cluster while their spinning motion enables them to roll about the middle ring's circumference. The four-microrobot cluster is too large for the spacing and jams between the two rings, forcing the middle ring to switch its rotation direction so it is the same as the outer ring. Although the jamming does not happen as soon as the microrobots begin to spin, it offers another level of control

over the ring’s rotation behavior; we can program the ring’s rotation behavior by exploiting physical jamming and fluidic torque while tuning the number of microrobots in each of the spatial regions, the field frequency, the number of rings and their size.

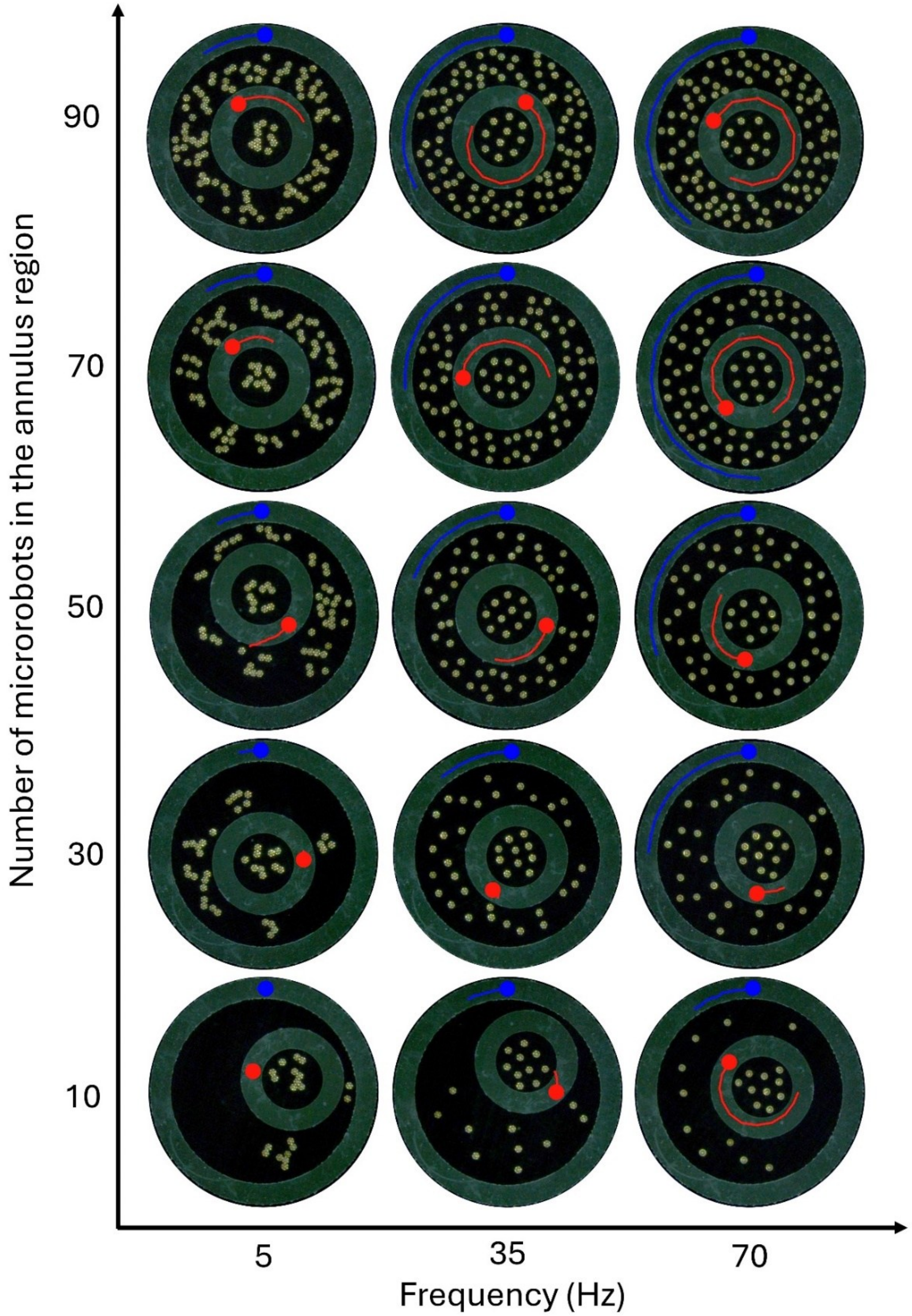

Fig. S2. Rotation visualization of concentric rings with 10 microrobots in the center region. Rotation is shown for two concentric rings during the final 3.3s of each experiment across a parameter space of magnetic field frequency and number of microrobots in the annulus region.

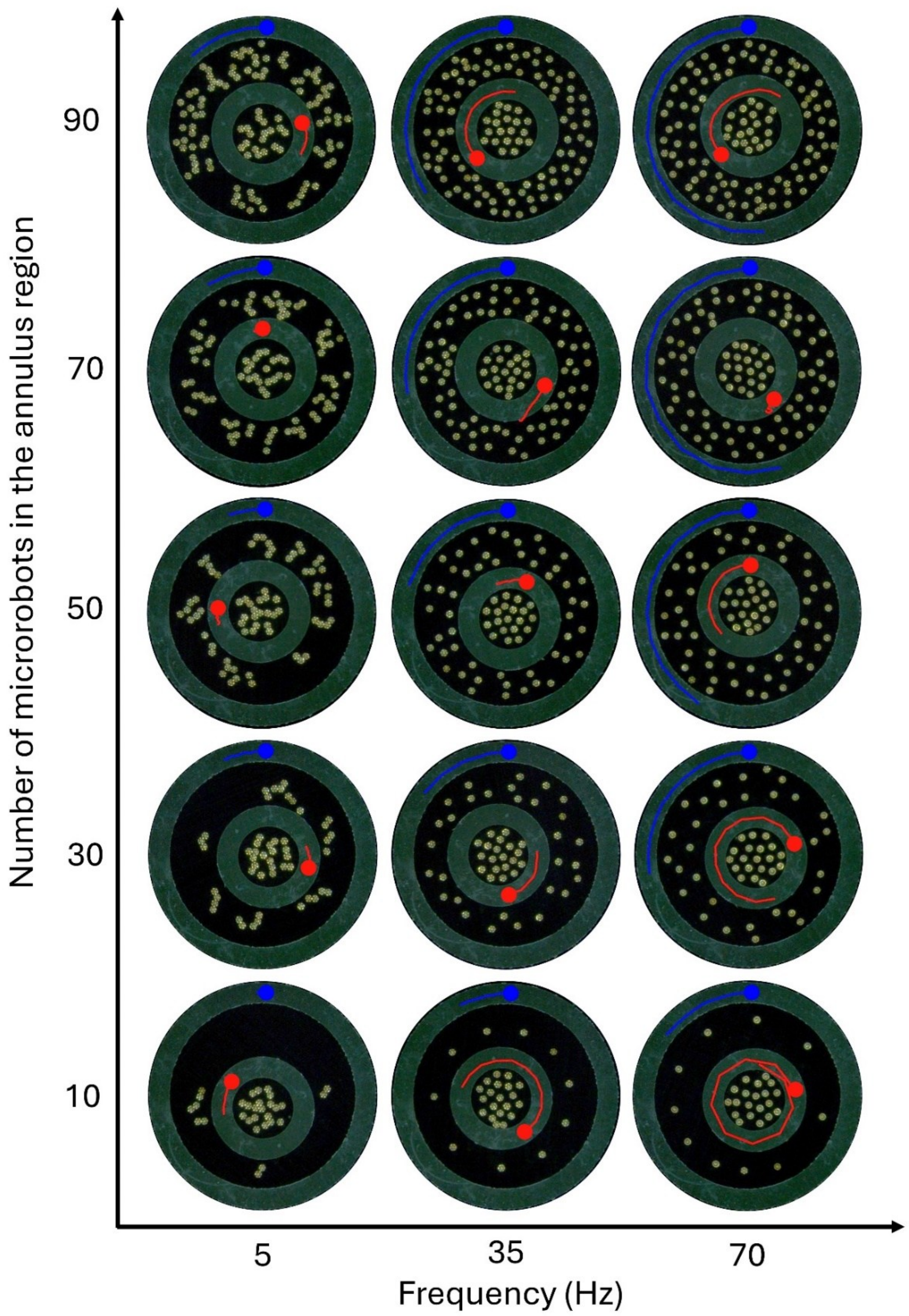

Fig. S3. Rotation visualization of concentric rings with 20 microrobots in the center region. Rotation is shown for two concentric rings during the final 3.3s of each experiment across a parameter space of magnetic field frequency and number of microrobots in the annulus region.

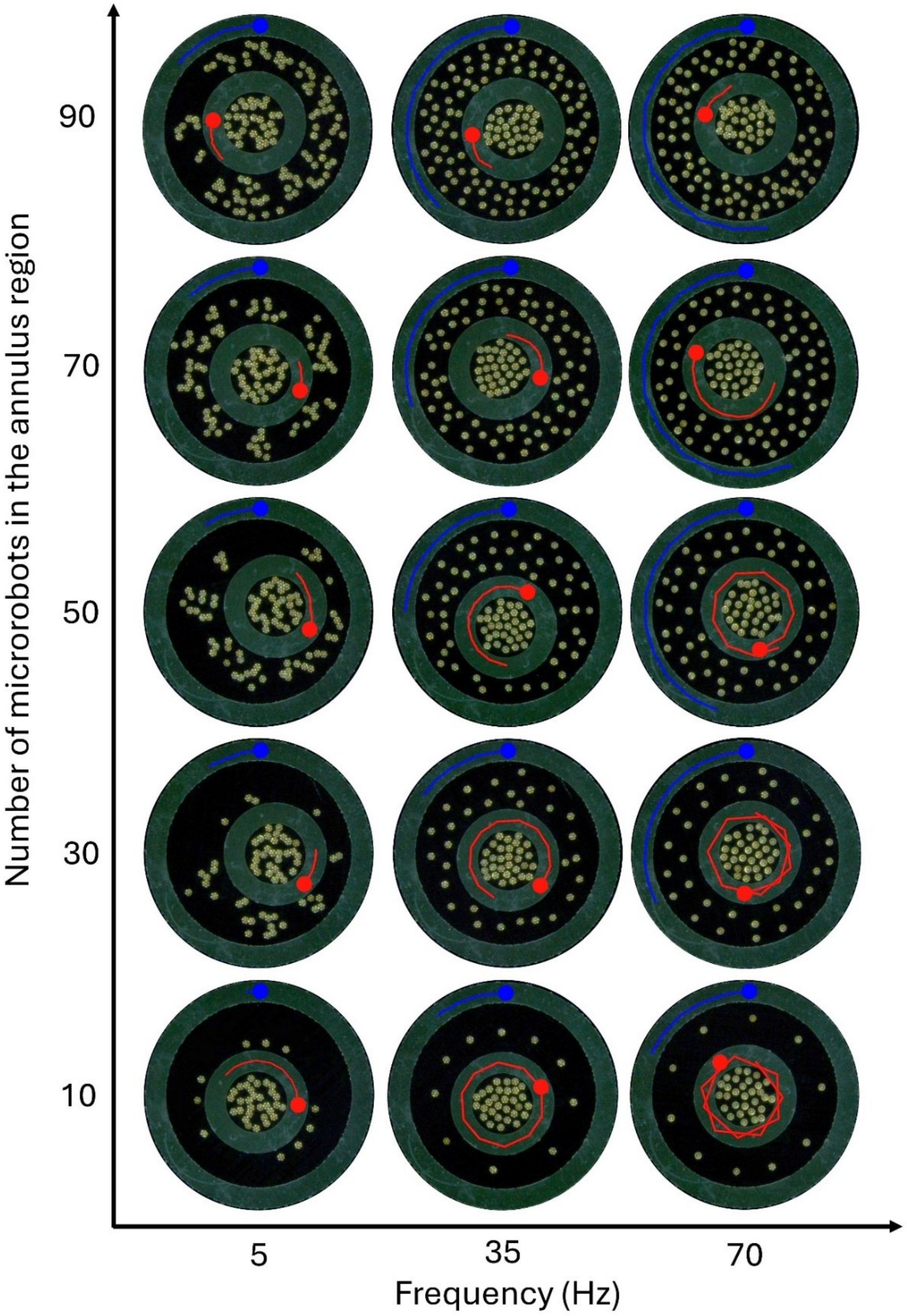

Fig. S4. Rotation visualization of concentric rings with 30 microrobots in the center region. Rotation is shown for two concentric rings during the final 3.3s of each experiment across a parameter space of magnetic field frequency and number of microrobots in the annulus region.

Model for microrobots

We simulated our microrobot collective to better understand the main factors that cause the fluidic torque that enables concentric rings to rotate in the same or opposite directions. The model used for simulations was adapted from (29) and modified to include the interactions between the microrobots and the passive rings. The passive rings exert a hydrodynamic repulsion on the microrobots (assumed to be similar in form to the hydrodynamic repulsion between any two microrobots). The model that is used to simulate the behaviors of the passive rings is described in the Model for passive rings Section. The modified model for the microrobots is as follows:

If the center-center distance $r_{ji} >$ lubrication threshold (=315 µm, or 2.1 R)

$$\frac{d\mathbf{r_i}}{dt} = \sum_{j\neq i}(6\pi\mu R)^{-1}\left(F_{mag-on,i,j}(r_{ji},\phi_{ji}) + F_{cap,i,j}(r_{ji},\phi_{ji}) + \frac{\rho\omega_i^2 R^7}{{r_{ji}}^3}\right)\cdot\hat{\mathbf{r}}_{\mathbf{ji}} + \sum_{j\neq i}\left(\frac{F_{mag-off,i,j}(r_{ji},\phi_{ji})}{6\pi\mu R} - \frac{R^3\omega_i}{{r_{ji}}^2}\right)\cdot\hat{\mathbf{r}}_{\mathbf{ji}}\times\hat{\mathbf{z}} + \sum_{j\neq i}\frac{\rho{\omega_i}^2R^7}{6\pi\mu R}\cdot\left(\left(\frac{1}{{d_{toLeft}}^3} - \frac{1}{{d_{toRight}}^3}\right)\cdot\hat{\mathbf{x}} + \left(\frac{1}{{d_{toBottom}}^3} - \frac{1}{{d_{toTop}}^3}\right)\cdot\hat{\mathbf{y}}\right) + \sum_{k=1,2}\boldsymbol{F}_{\boldsymbol{ik}}^{\boldsymbol{ring}}, \quad i = 1, 2, \ldots \tag{2}$$

$$\frac{d\alpha_i}{dt} = \frac{mB\sin(\theta-\alpha_i)}{8\pi\mu R^3} + \sum_{j\neq i}\frac{T_{mag-d,i,j}(r_{ji},\phi_{ji}) + T_{cap,i,j}(r_{ji},\phi_{ji})}{8\pi\mu R^3}, \quad i = 1,2,\ldots \tag{3}$$

$$\mathbf{B}(t) = \left(B_{x_0}\cos(\Omega_x t) + B_{x_1}\right)\cdot\hat{\mathbf{x}} + \left(B_{y_0}\cos(\Omega_y t) + B_{y_1}\right)\cdot\hat{\mathbf{y}}, \tag{4}$$

where $\mathbf{r_i}$ and $\mathbf{r_j}$ are the position vectors of microrobots *i* and *j*;

$\mathbf{r_{ji}} = \mathbf{r_i} - \mathbf{r_j}$ is the vector pointing from the center of microrobot j to the center of microrobot i;

α_i and α_j are the orientations of microrobots *i* and *j*;

d is the edge-edge distance between two microrobots;

ϕ_{ji} is the angle of dipole moment with respect to $\mathbf{r_{ji}}$. It is assumed to be the same for both microrobots, as $\phi_{ji} = \phi_i = \phi_j$;

ω_i is the instantaneous spin speed of the microrobots;

$B = |\mathbf{B}|$ is the magnetic field strength (10 mT);

$\theta = \arctan(B_y/B_x)$ is the orientation of the external magnetic field;

Ω_x and Ω_y are the oscillation frequencies of the x and y component of the external magnetic field, respectively;

R is the radius of each microrobot (150 µm);

μ is the dynamic viscosity of water (10^{-3} Pa·s);
ρ is the density of water (10^3 kg/m^3);
m is the magnetic dipole moment of the microrobots (10^{-8} A·m^2);
$F_{mag-on,i,j}$ and $F_{mag-off,i,j}$ are the magnetic dipole force on and off the center-to-center axis, respectively, and they are functions of r_{ji} and ϕ_{ji};
$T_{mag-d,i,j}$ is the magnetic dipole torque, and it is a function of r_{ji} and ϕ_{ji};
$F_{cap,i,j}$ is the capillary force, and it is a function of r_{ji} and ϕ_{ji} and embeds the symmetry of a micro-disk;
$T_{cap,i,j}$ is the capillary torque, and it is a function of r_{ji} and ϕ_{ji} and embeds the symmetry of a micro-disk;
d_{toLeft}, $d_{toRight}$, $d_{toBottom}$, and d_{toTop} are the distances of a microrobot to the four sides of the physical boundary;
$\boldsymbol{F}_{ik}^{ring}$ is the force exerted by the passive rings on the micro-disks (see the section on model for passive disks below for more details).

If the center-center distance $r_{ji} <$ lubrication threshold (=315 μm, or 2.1 R) and $r_{ji} \geq$ 300 or $2R$,

$$\mu \frac{d\mathbf{r_i}}{dt} = \sum_{j\neq i} A\left(\frac{d_{ji}}{R}\right)\left(F_{mag-on,\,i,j}(r_{ji}, \varphi_{ji}) + F_{cap,\,i,j}(r_{ji}, \varphi_{ji}) + \frac{\rho\omega^2 R^7}{r_{ji}^3}\right)\hat{\mathbf{r}}_{\mathbf{ji}} + \sum_{j\neq i} B\left(\frac{d_{ji}}{R}\right) F_{mag-off,\,i,j}(r_{ji}, \varphi_{ji})\,\hat{\mathbf{r}}_{\mathbf{ji}} \times \hat{\mathbf{z}} + \sum_{j\neq i} C\left(\frac{d_{ji}}{R}\right) mB\sin(\theta - \alpha_i)\,\hat{\mathbf{r}}_{\mathbf{ji}} \times \hat{\mathbf{z}} + \frac{\rho\omega_i^2 R^7}{6\pi R}\left(\left(\frac{1}{d_{toLeft}^3} - \frac{1}{d_{toRight}^3}\right)\hat{\mathbf{x}} + \left(\frac{1}{d_{toBottom}^3} - \frac{1}{d_{toTop}^3}\right)\hat{\mathbf{y}}\right) + \sum_{k=1,2} \boldsymbol{F}_{ik}^{ring},\ i = 1,2,\ldots \tag{5}$$

$$\mu \frac{d\alpha_i}{dt} = G\left(\frac{d_{smallest}}{R}\right) mB\sin(\theta - \alpha_i) + \sum_{j\neq i} G\left(\frac{d_{ji}}{R}\right) T_{mag-d,i,j}(r_{ji}, \varphi_{ji}) + T_{cap,i,j}(r_{ji}, \varphi_{ji}),\ i = 1,2,\ldots \tag{6}$$

$$\mathbf{B}(t) = \left(B_{x_0}\, cos(\Omega_x t) + B_{x_1}\right)\cdot\hat{\mathbf{x}} + \left(B_{y_0}\, cos(\Omega_y t) + B_{y_1}\right)\cdot\hat{\mathbf{y}}\,, \tag{7}$$

where the coefficients $A(x), B(x), C(x) and\ G(x)$ are lubrication coefficients.

If the center-center distance $r_{ji} < 300$ or $2R$, a repulsion term is added to the force equation,

$$\mu \frac{d\mathbf{r_i}}{dt} = \sum_{j\neq i} A(\varepsilon)\left(F_{mag-on,\,i,j}(2R, \varphi_{ji}) + F_{cap,\,i,j}(2R, \varphi_{ji}) + \frac{\rho\omega^2 R^7}{r_{ji}^3}\right)\hat{\mathbf{r}}_{\mathbf{ji}} + \sum_{j\neq i} \frac{F_{wallRepulsion}}{6\pi R}\cdot\frac{-d_{ji}}{R}\hat{\mathbf{r}}_{\mathbf{ji}} + \sum_{j\neq i} B(\varepsilon) F_{mag-off,\,i,j}(2R, \varphi_{ji})\,\hat{\mathbf{r}}_{\mathbf{ji}}\times\hat{\mathbf{z}} + \sum_{j\neq i} C(\varepsilon)\, mB\sin(\theta-\alpha_i)\,\hat{\mathbf{r}}_{\mathbf{ji}}\times\hat{\mathbf{z}} + \frac{\rho\omega_i^2 R^7}{6\pi R}\left(\left(\frac{1}{d_{toLeft}^3}-\frac{1}{d_{toRight}^3}\right)\hat{\mathbf{x}} + \left(\frac{1}{d_{toBottom}^3}-\frac{1}{d_{toTop}^3}\right)\hat{\mathbf{y}}\right) + \sum_{k=1,2} \boldsymbol{F}_{\boldsymbol{ik}}^{\boldsymbol{ring}},\ i = 1,2,\ldots \tag{8}$$

$$\mu\frac{d\alpha_i}{dt} = G(\varepsilon) mB\sin(\theta-\alpha_i) + \sum_{j\neq i} G(\varepsilon) T_{mag-d,i,j}(2R,\ \varphi_{ji}) + T_{cap,i,j}(2R,\ \varphi_{ji}),\ i = 1,2,\ldots \tag{9}$$

$$\mathbf{B}(t) = \left(B_{x_0}\, cos(\Omega_x t) + B_{x_1}\right)\cdot\hat{\mathbf{x}} + \left(B_{y_0}\, cos(\Omega_y t) + B_{y_1}\right)\cdot\hat{\mathbf{y}}\,, \tag{10}$$

where ε is a small number (10^{-10} μm/R); $F_{wallRepulsion}$ is set to be 10^{-7} N.

Model for passive rings

The passive rings exert a hydrodynamic repulsion on the microrobots (assumed to be similar in form to the hydrodynamic repulsion between any two microrobots). The microrobots generate an azimuthal flow field that causes the rings to rotate. In the model, we assume that the microrobot collective is homogeneously spread within the available space and the net azimuthal flow at the circumference of the passive ring is approximated using the minimum flow generated by a single microrobot in the collective (Fig. S5) multiplied by the total number of microrobots times a constant. The constant is the same for both the rings for all the experiments and is equal to 0.1. We add the effects of the microrobots inside and outside a passive ring to get the final rotation speed of the ring. We neglect the effect of the rotation of the passive rings on the microrobots. We also assume the effect of one passive ring on the other to be minimal due to the symmetry of the system. The interactions between the passive rings and the microrobots is modelled as follows: If the distance between a microrobot's center and the nearest point on the passive ring circumference d_{ki} > lubrication threshold (=165 μm, or 1.1 R)

$$\boldsymbol{F}_{ik}^{ring} = \sum_{k=1,2} \left(\frac{\rho {\omega_i}^2 R^7}{6\pi\mu R} \cdot \left(\frac{1}{d_{ki}^3} \right) \cdot \hat{\mathbf{r}}_{\mathbf{ki}} \right), \tag{11}$$

$$\frac{d\alpha_k^{\mathrm{ring}}}{dt} = c_k N_k^{inside} \cdot \min_{\forall i_{inside}} \left(\left| -\frac{R^3 \omega_i}{d_{ik}^2} \cdot \hat{\boldsymbol{r}}_{\boldsymbol{ik}} \times \hat{\boldsymbol{z}} \right| \right) \frac{sgn(\omega_i)}{R_k^{ring}} + c_k N_k^{outside} \cdot \min_{\forall i_{outside}} \left(\left| -\frac{R^3 \omega_i}{d_{ik}^2} \cdot \hat{\boldsymbol{r}}_{\boldsymbol{ik}} \times \hat{\boldsymbol{z}} \right| \right) \frac{sgn(\omega_i)}{R_k^{ring}}, \quad k = 1, 2 \tag{12}$$

α_k^{ring} is the orientation of the $\mathrm{k^{th}}$ ring;
$\hat{\boldsymbol{r}}_{\boldsymbol{ik}}$ is the unit vector from center of the ith microrobot to the nearest point on the circumference of the kth passive ring;
d_{ik} is the distance of the center of the ith microrobot from the nearest point on the circumference of the kth passive ring;
R_k^{ring} is the radius of the kth ring;
$sgn(\omega_i) = \frac{\omega_i}{|\omega_i|}$ is the sign of the angular velocity of the ith microrobot;
$c_k = 0.1$ is a constant and is the same for both the passive rings;
$N_k^{inside}, N_k^{outside}$ are the total number of microrobot inside and outside each of the passive rings;

If the distance between micro-disk center and the nearest point on the passive ring circumference d_{ki} < lubrication threshold (=165 μm, or 1.1 R) and $d_{ki} > 150$ μm or R

$$\boldsymbol{F}_{ik}^{ring} = \sum_{k=1,2} A\left(\frac{d_{ki}}{R}\right) \left(\frac{\rho {\omega_i}^2 R^7}{6\pi\mu R} \cdot \left(\frac{1}{d_{ki}^3} \right) \cdot \hat{\mathbf{r}}_{\mathbf{ki}} \right), \tag{13}$$

If the distance between a microrobot's center and the nearest point on the passive ring circumference $d_{ki} < 150$ μm, or R the repulsion force is modified to prevent the overlap between the microrobot and the ring,

$$\boldsymbol{F}_{\boldsymbol{ik}}^{\boldsymbol{ring}} = \sum_{k=1,2} \left(\frac{F_{wallRepulsion}}{6\pi\mu R} \cdot \left(\frac{-d_{ki}}{R} \right) \cdot \hat{\mathbf{r}}_{\mathbf{ki}} \right), \tag{14}$$

$F_{wallRepulsion}$ is set to 10^{-7} N (the same value as that used in the model for microrobots). The model for angular velocity of the passive rings remains unchanged under different distance regimes because at any instance, the majority of the microrobots are at a distance $d_{ki} >$ lubrication threshold. Thus, the microrobot(s) generating the minimum value of the flow at the circumference of the passive rings is always at a distance $d_{ki} >$ lubrication threshold.

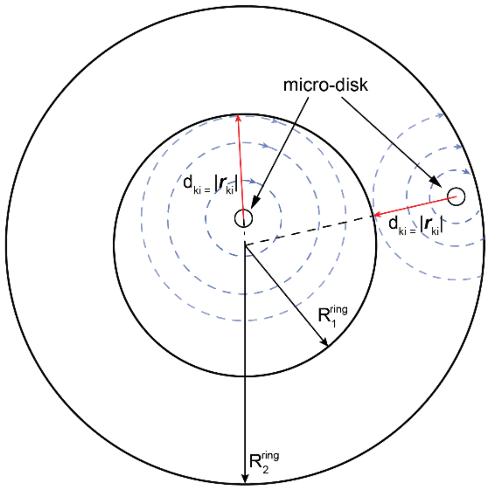

Fig. S5. The schematic for the model of passive rings. The microrobots outside and inside a passive ring generate an azimuthal flow field that causes the ring to rotate. The shortest distance between a microrobot and a passive ring is used for the calculation of the flow field. The net flow field generated by the microrobot (inside or outside) at the circumference of a passive ring is approximated by the smallest value of the flow generated by a single microrobot in the collective and the value is multiplied by the total number of microrobots (inside or outside) and a constant. The constant is 0.1 for both the passive rings for all the experiments.

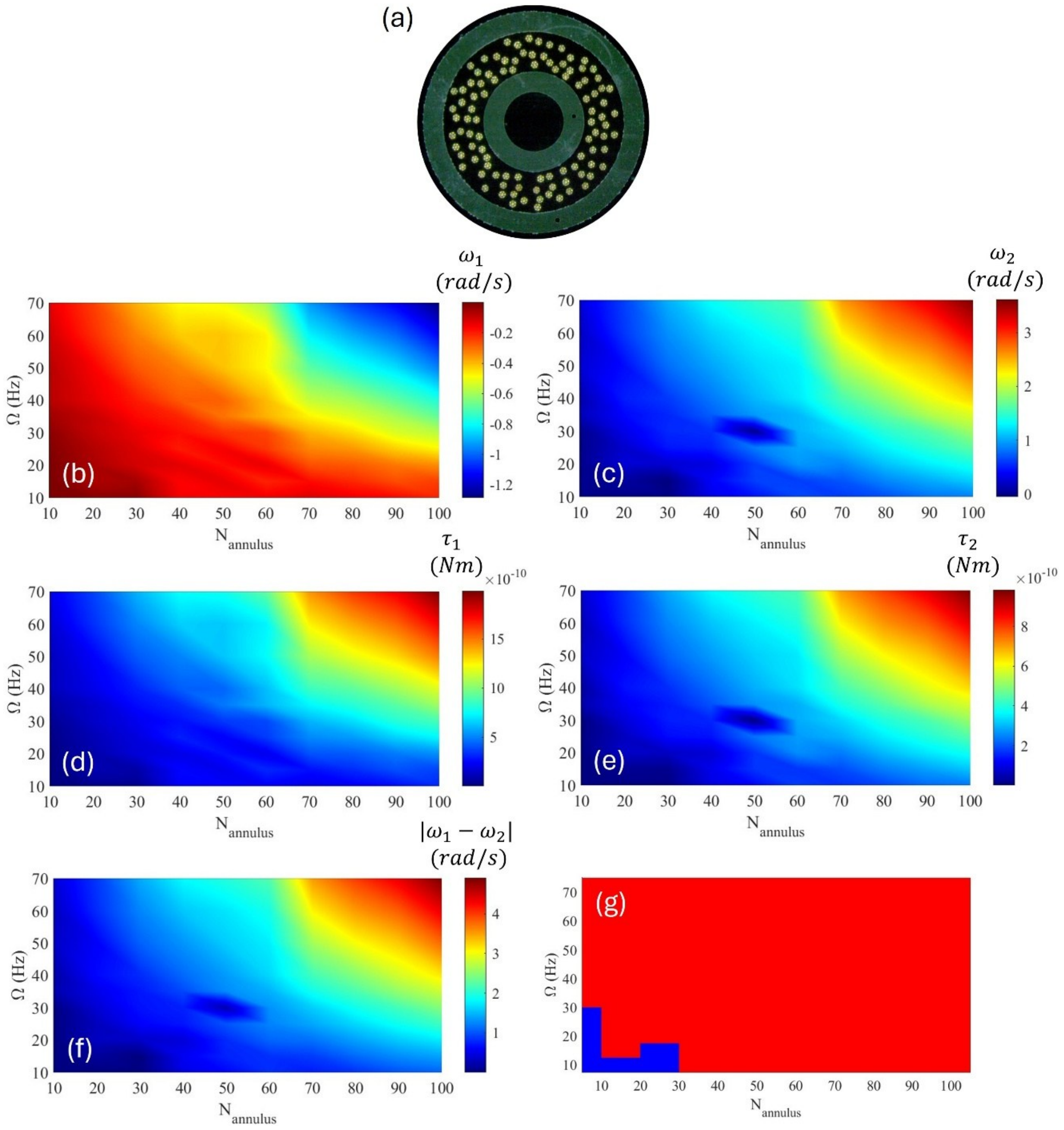

Fig. S6. Two concentric rings with microrobots in the annulus region. (a) Representative image of two concentric ring structures with 100 microrobots in the annulus region. **(b)** Angular velocity of outer ring structure. **(c)** Angular velocity of the inner ring structure. **(d)** Applied torque at inner boundary of the outer ring structure. **(e)** Applied torque at outer boundary of the inner ring structure. **(f)** Absolute difference between angular velocities of outer and inner ring structures. **(g)** Map of regions in which rings rotate in the same direction (blue) and in opposite directions (red); the blue regions at the bottom left of the $N_{annulus}$-Ω parameter space are due to uneven torque distribution about the perimeter of the inner and outer ring which may cause both rings to rotate in the same direction when there is a low magnetic field frequency and a low number of microrobots in the annulus region.

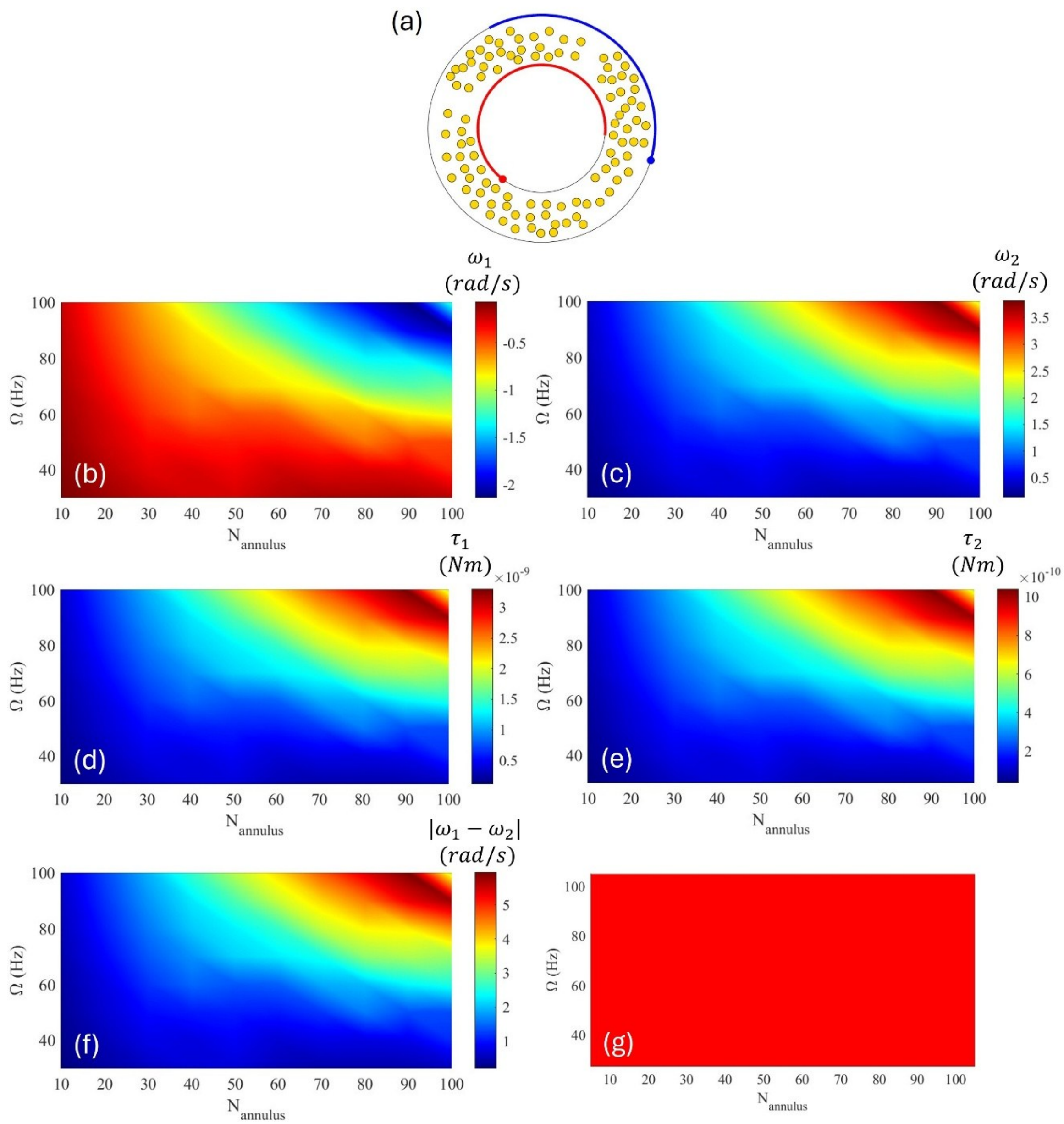

Fig. S7. Simulations of two concentric rings with microrobots in annulus region. (a) Representative image of two concentric ring structures with 60 simulated microrobots in the annulus region. **(b)** Angular velocity of outer ring structure. **(c)** Angular velocity of the inner ring structure. **(d)** Applied torque at inner boundary of the outer ring structure. **(e)** Applied torque at outer boundary of the inner ring structure. **(f)** Absolute difference between angular velocities of outer and inner ring structures. **(g)** Map of regions in which rings rotate in the same direction (blue) and in opposite directions (red).

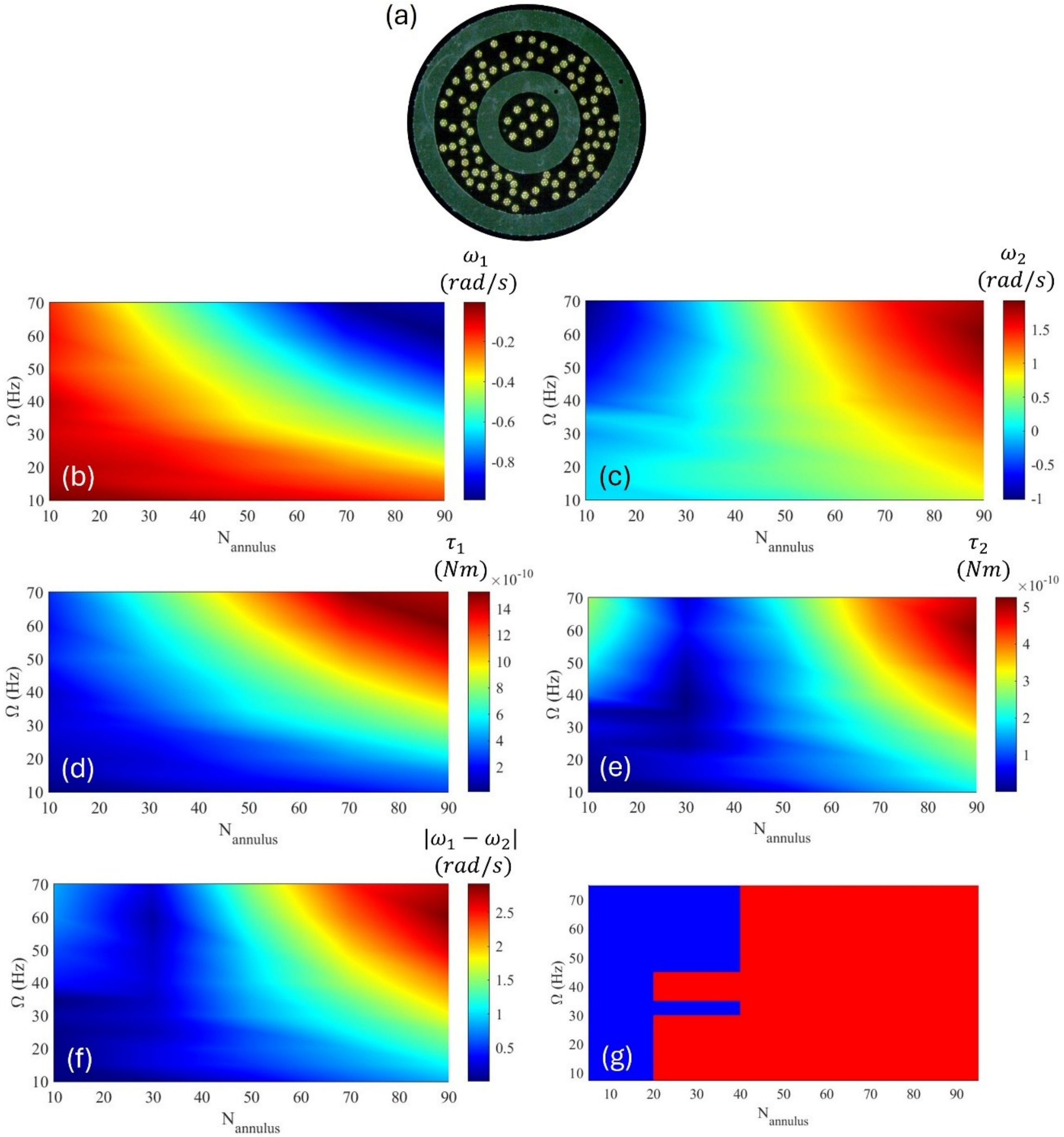

Fig. S8. Two concentric rings with 10 microrobots in the center region. (a) Representative image of two concentric ring structures with 10 microrobots in the center region and 90 in the annulus region. **(b)** Angular velocity of outer ring structure. **(c)** Angular velocity of inner ring structure. **(d)** Applied torque at inner boundary of the outer ring structure. **(e)** Applied torque at outer boundary of the inner ring structure. **(f)** Absolute difference between angular velocities of outer and inner ring structures. **(g)** Map of regions in which rings rotate in the same direction (blue) and in opposite directions (red).

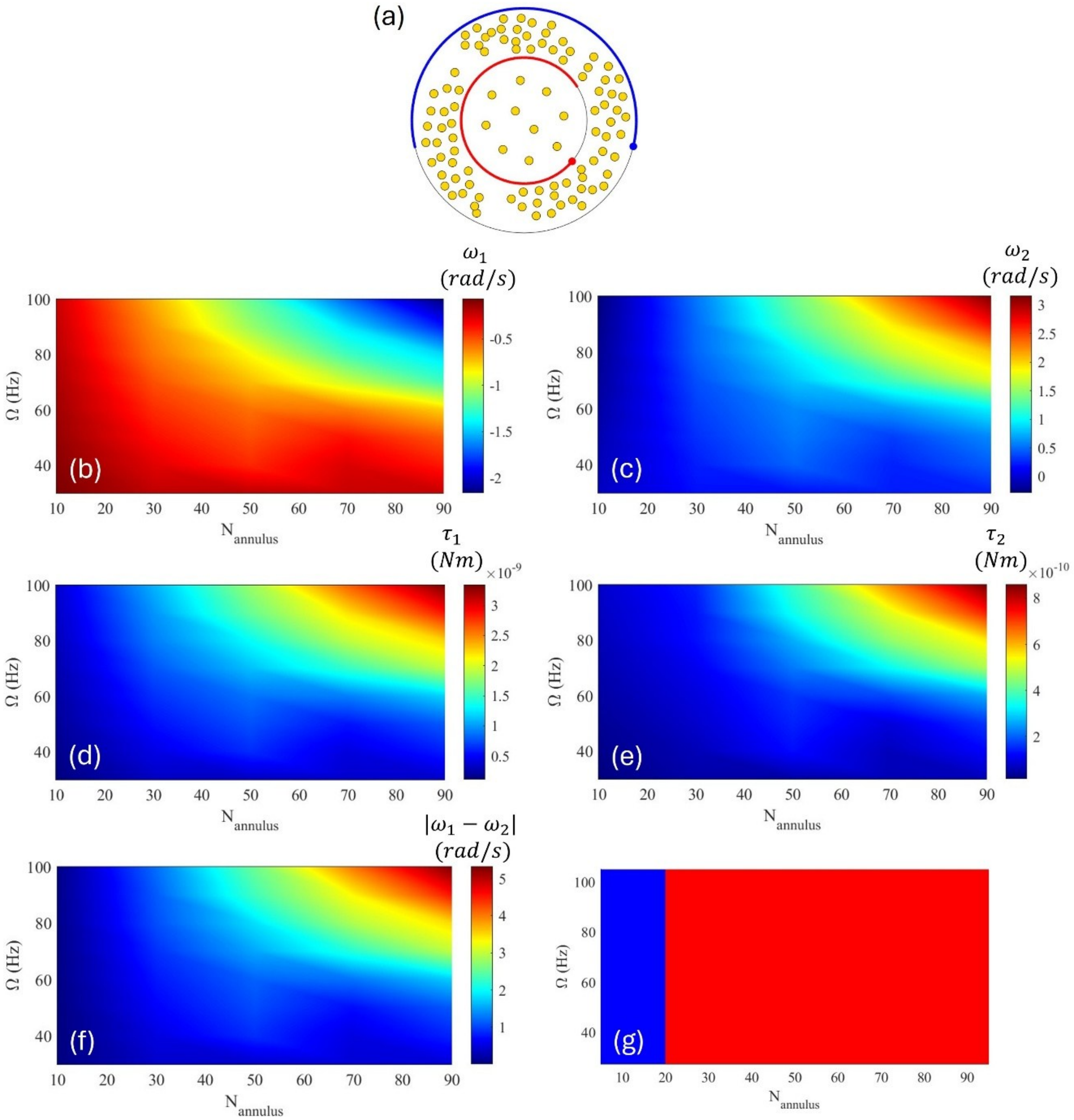

Fig. S9. Simulations of two concentric rings with 10 microrobots in the center region. (a) Representative image of two concentric ring structures with 10 microrobots in the center region and 90 in the annulus region. **(b)** Angular velocity of outer ring structure. **(c)** Angular velocity of inner ring structure. **(d)** Applied torque at inner boundary of the outer ring structure. **(e)** Applied torque at outer boundary of the inner ring structure. **(f)** Absolute difference between angular velocities of outer and inner ring structures. **(g)** Map of regions in which rings rotate in the same direction (blue) and in opposite directions (red).

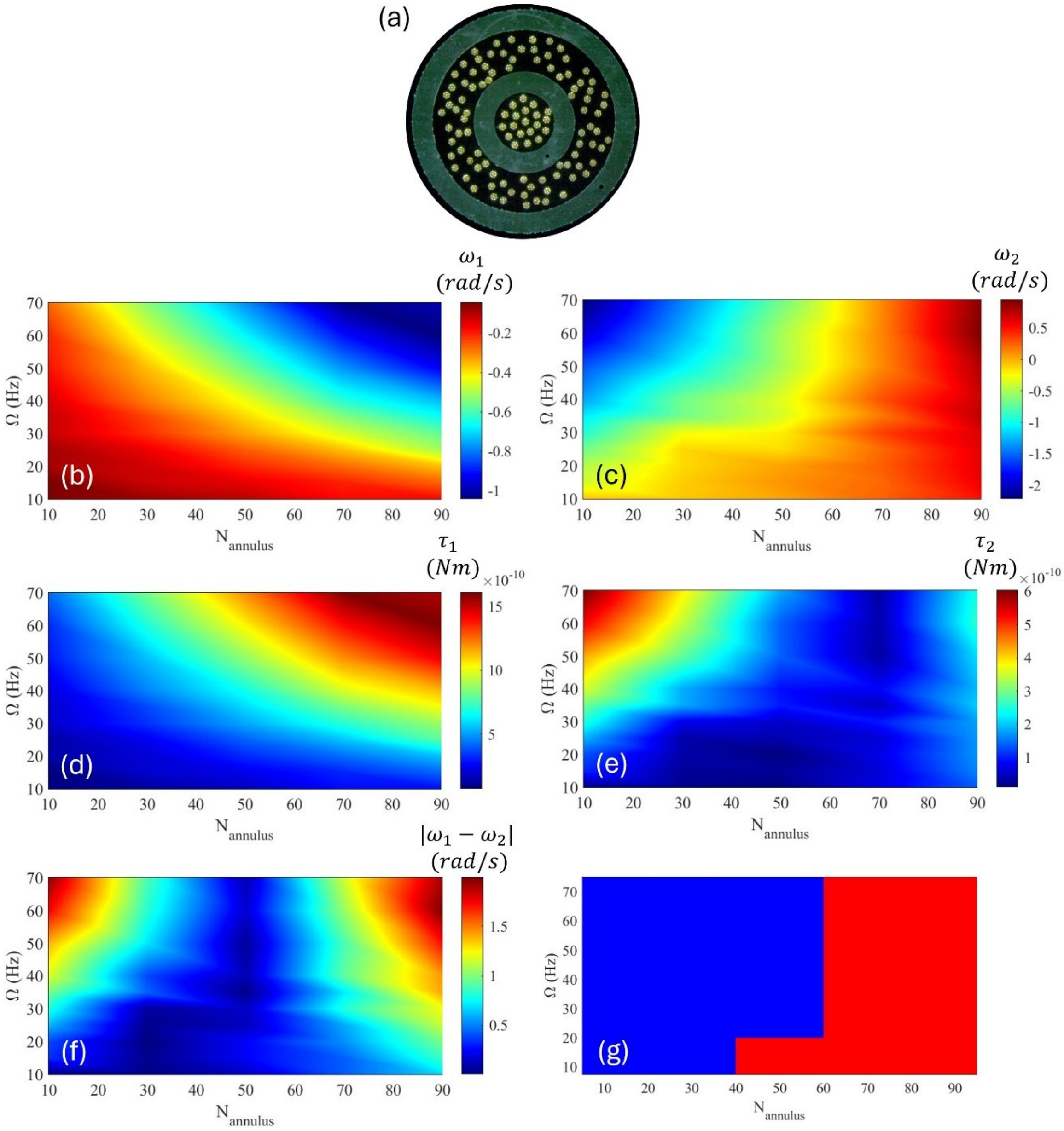

Fig. S10. Two concentric rings with 20 microrobots in the center region. (a) Representative image of two concentric ring structures with 30 microrobots in the center region and 90 in the annulus region. **(b)** Angular velocity of outer ring structure. **(c)** Angular velocity of the inner ring structure. **(d)** Applied torque at inner boundary of the outer ring structure. **(e)** Applied torque at outer boundary of the inner ring structure. **(f)** Absolute difference between angular velocities of outer and inner ring structures. **(g)** Map of regions in which rings rotate in the same direction (blue) and in opposite directions (red).

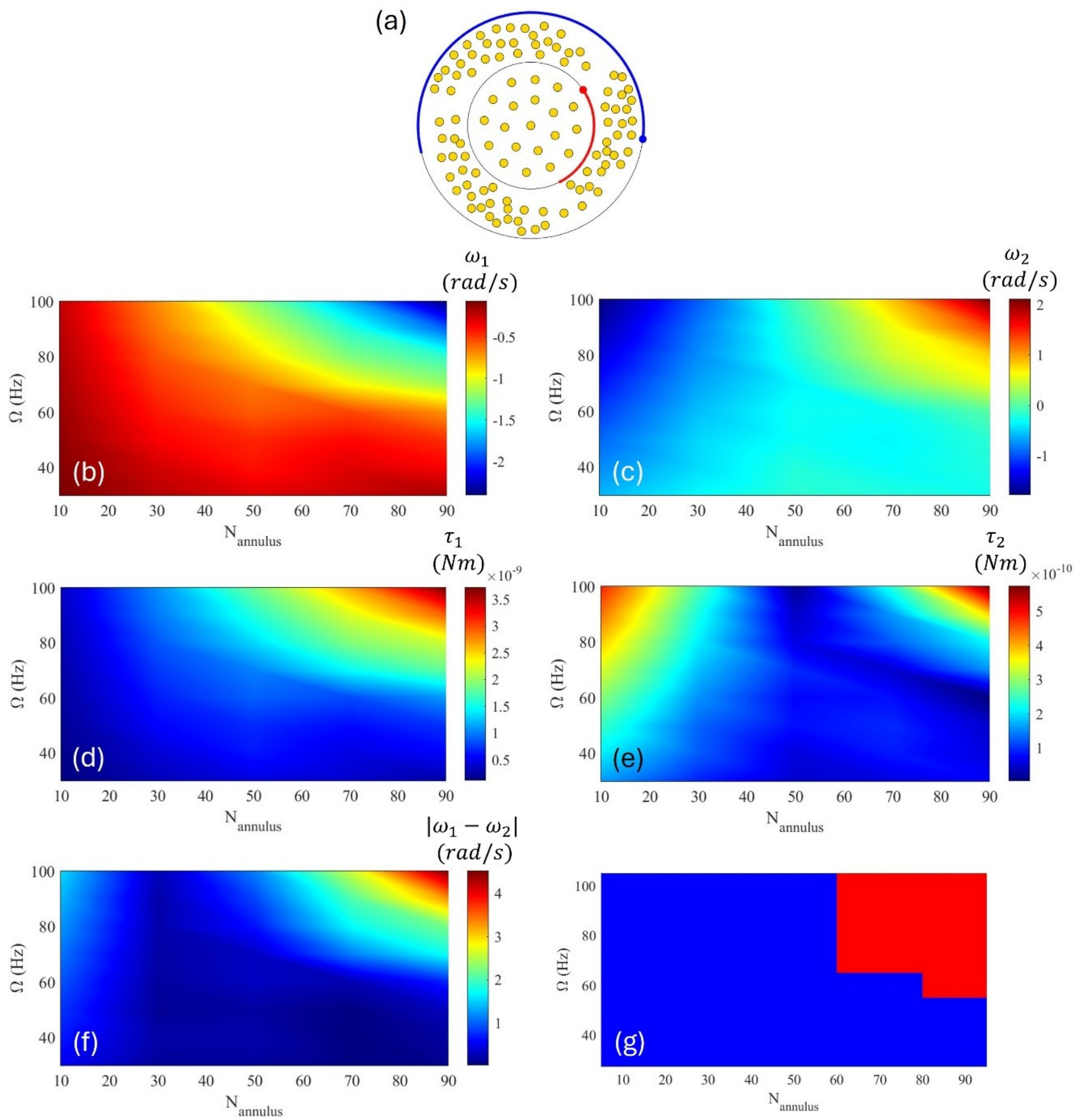

Fig. S11. Simulations of two concentric rings with 20 microrobots in the center region. (a) Representative image of two concentric ring structures with 20 microrobots in the center region and 90 in the annulus region. **(b)** Angular velocity of outer ring structure. **(c)** Angular velocity of the inner ring structure. **(d)** Applied torque at inner boundary of the outer ring structure. **(e)** Applied torque at outer boundary of the inner ring structure. **(f)** Absolute difference between angular velocities of outer and inner ring structures. **(g)** Map of regions in which rings rotate in the same direction (blue) and in opposite directions (red).

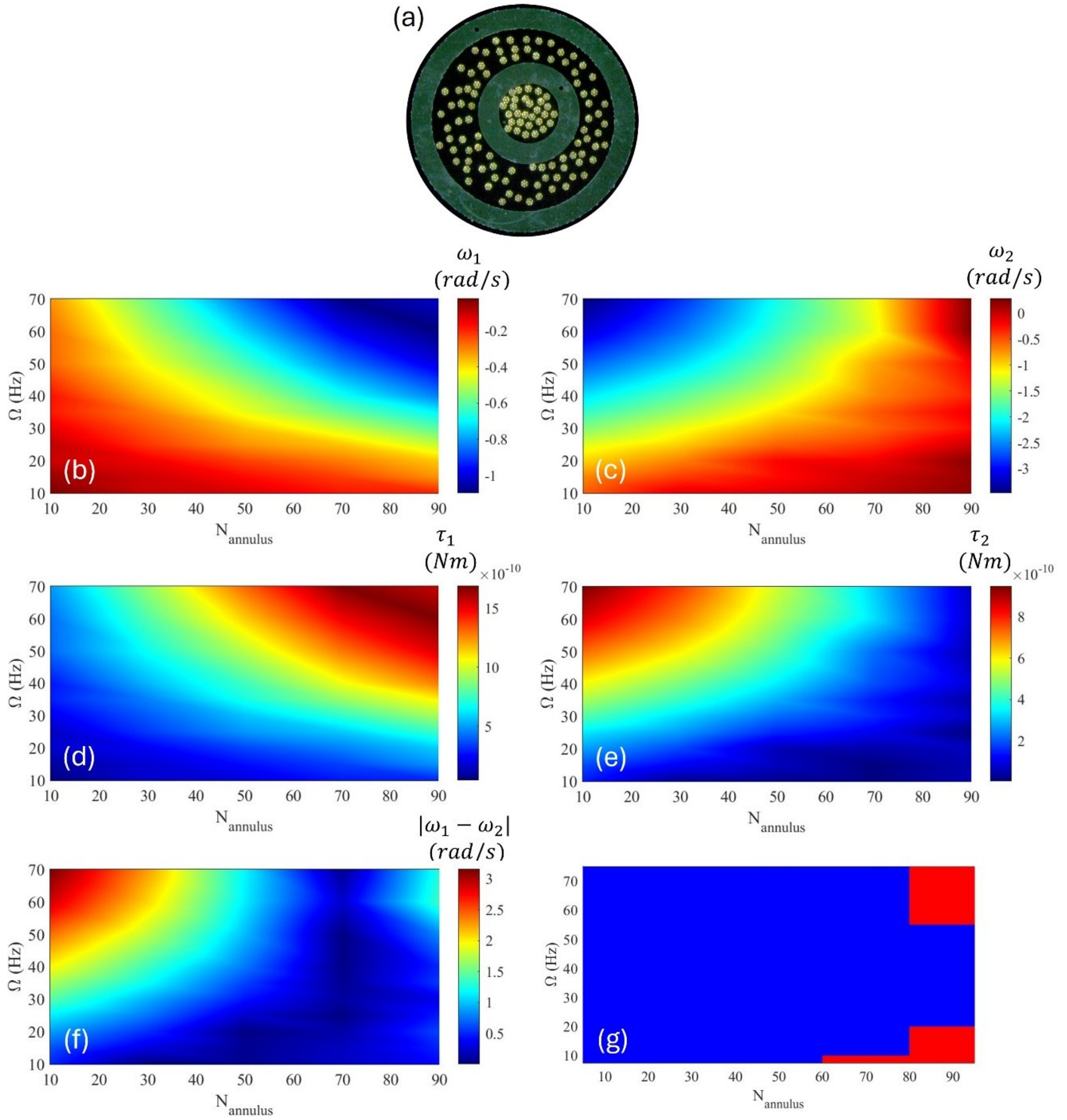

Fig. S12. Two concentric rings with 30 microrobots in the center region. (a) Representative image of two concentric ring structures with 30 microrobots in the center region and 90 in the annulus region. **(b)** Angular velocity of outer ring structure. **(c)** Angular velocity of the inner ring structure. **(d)** Applied torque at inner boundary of the outer ring structure. **(e)** Applied torque at outer boundary of the inner ring structure. **(f)** Absolute difference between angular velocities of outer and inner ring structures. **(g)** Map of regions in which rings rotate in the same direction (blue) and in opposite directions (red).

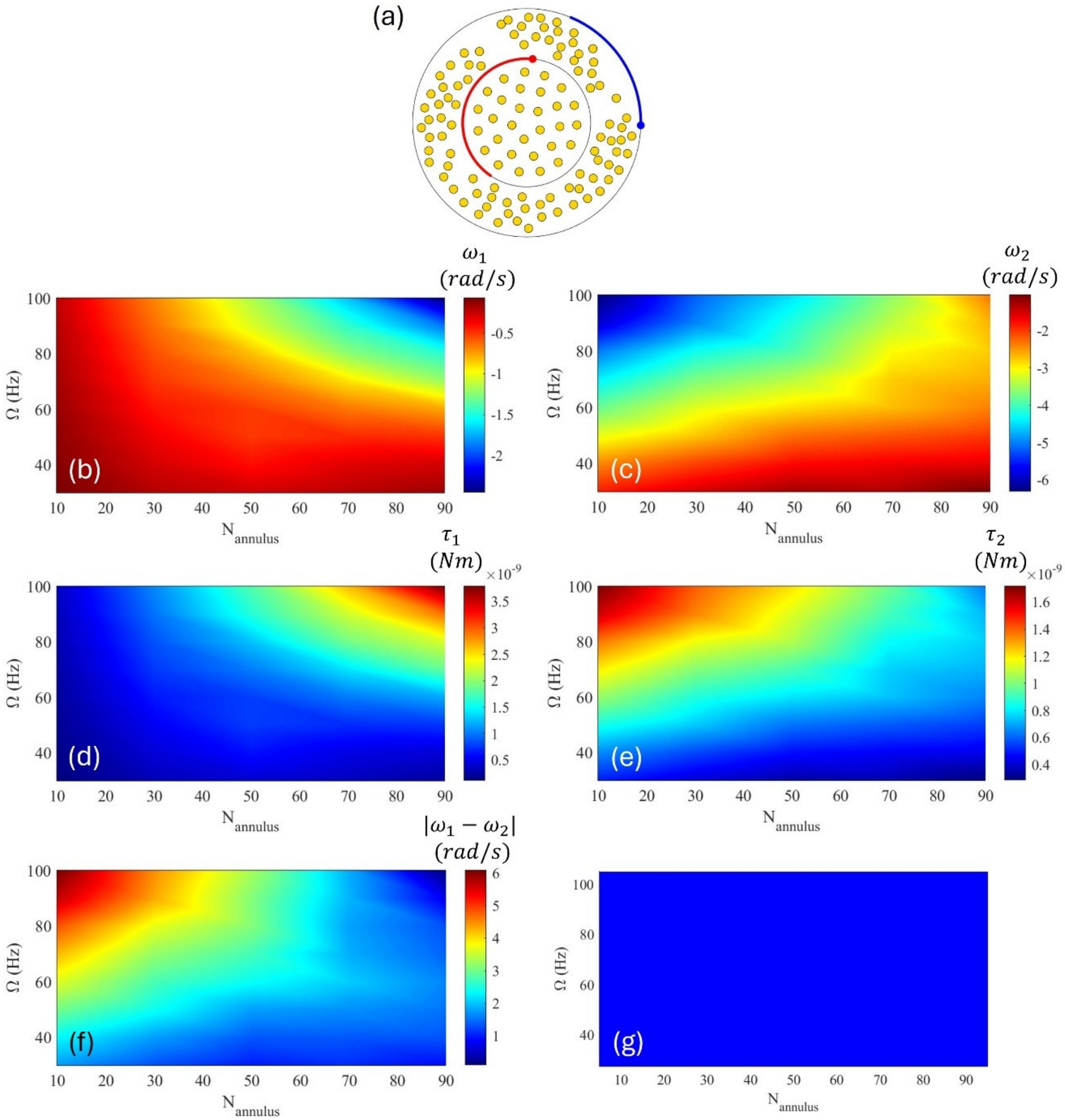

Fig. S13. Simulations of two concentric rings with 30 microrobots in the center region. (a) Representative image of two concentric ring structures with 30 microrobots in the center region and 90 in the annulus region. **(b)** Angular velocity of outer ring structure. **(c)** Angular velocity of the inner ring structure. **(d)** Applied torque at inner boundary of the outer ring structure. **(e)** Applied torque at outer boundary of the inner ring structure. **(f)** Absolute difference between angular velocities of outer and inner ring structures. **(g)** Map of regions in which rings rotate in the same direction (blue) and in opposite directions (red).

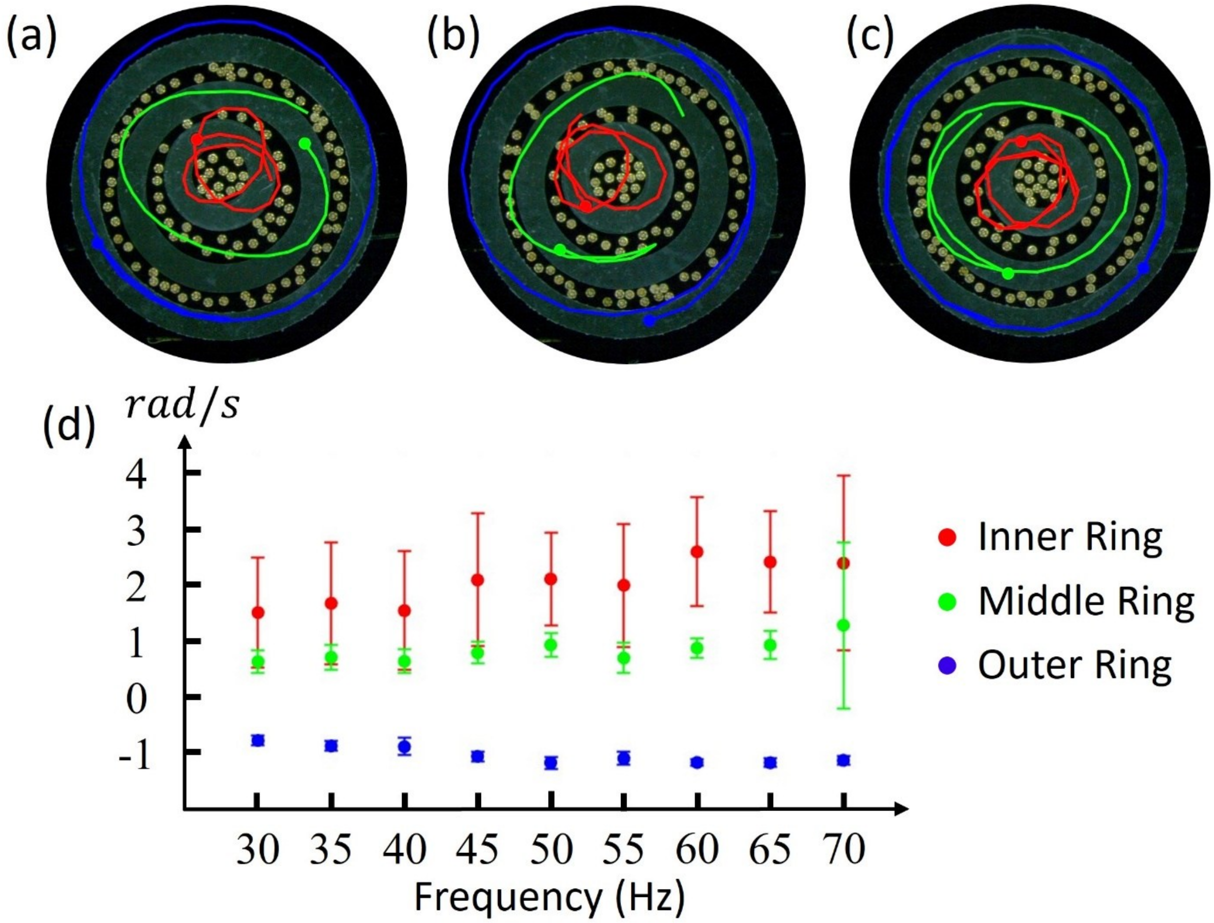

Fig. S14. Three concentric rings. Three concentric ring structures are actuated by 15 robots in the center region, 30 in the inner annulus region, and 60 in the outer annulus region at **(a)** $\Omega = 30$ Hz, **(b)** $\Omega = 40$ Hz, and **(c)** $\Omega = 50$ Hz. In (b), the middle ring's change in direction midway through the experiment shows how jamming between the microrobots in the small annulus region can enable the rings to rotate in the opposite direction than they otherwise would through the fluidic torque. The trajectories in (a-c) are shown for a 10-second experiment. **(d)** The angular velocity of the inner (red), middle (green), and outer (blue) ring structures is shown across various frequencies.

Description of experiments with gear-like systems

To explore how fluidic torque could be used with traditional mechanical force transfer, we designed the following experiments involving the actuation of gear-like structures: two- and three-gear systems, a gripper, and a rack-and-pinion-like system, as shown in Fig. S15. In the two- and three-gear experiments, a microrobot collective generates flows that rotate the gear in which it lies, and upon actuation, the gear rotates another gear through a combination of physical contact and capillary interactions. Each of the gears has a fixed center that enables them to rotate about a center point. Each gear has an open central region that encircles a fixed rod which has a small annulus of PDMS that encircles its perimeter and is level with the water line. The annulus region between the center PDMS and the central region of each gear is where the microrobots are placed. Contact between the center structure and the gear structure opens the possibility of irreversible attachment driven by attractive capillary interactions because of surface imperfections; therefore, we only show results when there is a high number of microrobots within the driven gear (40 microrobots were used in the experiments reported in the paper). This enables the annulus region to have a high density of microrobots which increases the fluidic drag on the inner perimeter of the gear so that it can consistently overcome the capillary interactions; however, in many cases it was possible to actuate these gear trains using a much lower number of microrobots. The microrobots must supply enough torque to rotate the mass of the second gear and overcome the hydrodynamic drag that results from its fluidic interaction with its center, the actuated gear, and its underside area in contact with the fluid. In the two-gear experiment (Fig. 15a), the two gears are fixed at their center and the one on the left is actuated with 40 microrobots so that it rotates clockwise; this causes the gear on the right side to rotate counterclockwise. In the three-gear experiment (Fig. S15b), there is one actuated gear which is made to rotate clockwise; the second gear (on the bottom) rotates counterclockwise, and a third gear (adjacent to the second gear) rotates clockwise. Longer sequences of gears could potentially be driven by increasing the frequency range at which the microrobots can spin; this would enable them to supply enough torque to overcome the additional fluidic drag and inter-structure capillary interactions that would oppose the motion of the larger system. We also actuated a gripper-like structure so that it would open a set of claws extruding from the gears (Fig. S15c). The gripper rotates between the open and close positions at a slower rate than the gears in Figs. S15a-b because the long extrusions from the center create a higher drag that must be overcome to rotate the two adjacent structures. In the rack-and-pinion-like experiment (Fig. S15d), the microrobots are placed within the gear and actuate it so that it moves along the length of the rack. In this setup, both the rack and the pinion are free-floating within a square arena; however, because of the boundary repulsion effect, the rack slightly rotates but remains close to the starting position. The pinion is actuated by eight microrobots and converts its rotational motion into translation by rolling along the rack. The pinion does not move away from the rack because of a combination of the capillary attraction and the arena boundary repulsion pushing it towards the center of the workspace. This experiment demonstrates how small-scale machines could exploit capillary interactions, so that structures maintain contact even when they are free-floating and convert collective rotation into the transport of a larger object.

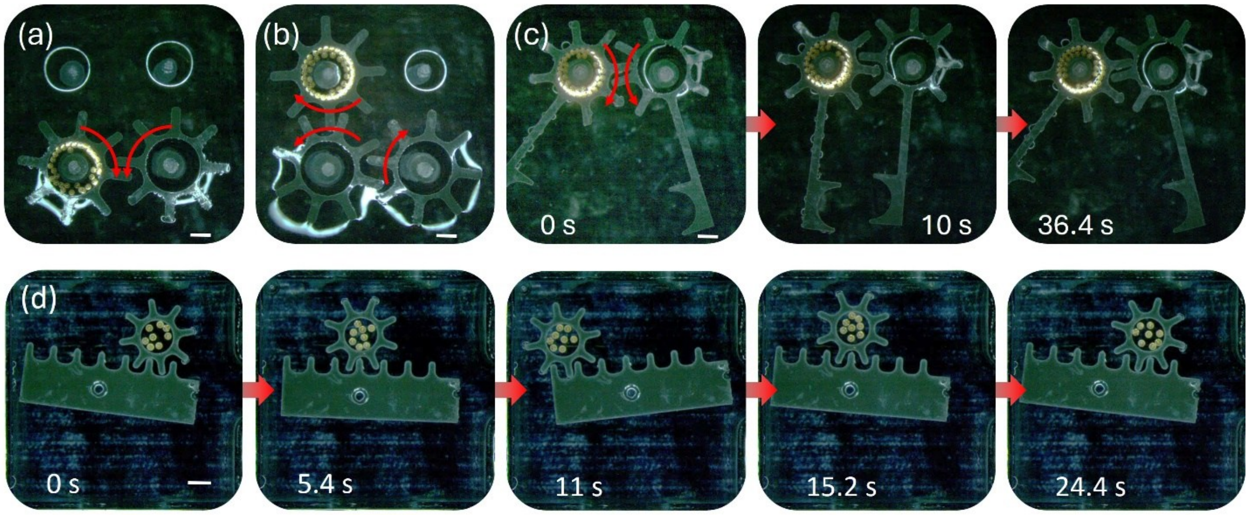

Fig. S15. Gear systems driven by microrobot collectives. (a) Microrobots are within a pinned gear that drives an adjacently pinned gear. **(b)** Microrobots are within a pinned gear that drives two pinned gears. **(c)** Microrobots driving a gripper structure. **(d)** Rack-and-pinion system in which eight microrobots inside the pinion drive it so it moves along the length of the rack.

Characterization of dynamic self-assembly experiments

When 15 non-concentric rings are each embedded with three microrobots, each concentric ring spins, and the rotational flows enable their dynamical self-assembly. As shown in Fig. S16, at lower frequencies there is insufficient hydrodynamic repulsion between the rings to overcome the capillary attraction between the rings and the magnetic attraction coming from the three microrobots within each ring structure. These are the same mechanisms that lead microrobot collectives to cluster at the lower frequencies. As shown in Fig. S17, the ring-structure collective follows the same trends as the microrobot collectives for the collective radius, especially at higher frequencies when all rings have disconnected from each other. Up to $\Omega = 25$ Hz, several of the rings are still attached to each other because of the attractive interactions. The microrobot collectives tend to break apart once $\Omega > 15$ because at this point the hydrodynamic repulsion becomes greater than the attractive capillary and magnetic interactions; this is a lower frequency than for the ring structures because the ring structures enable higher capillary attraction.

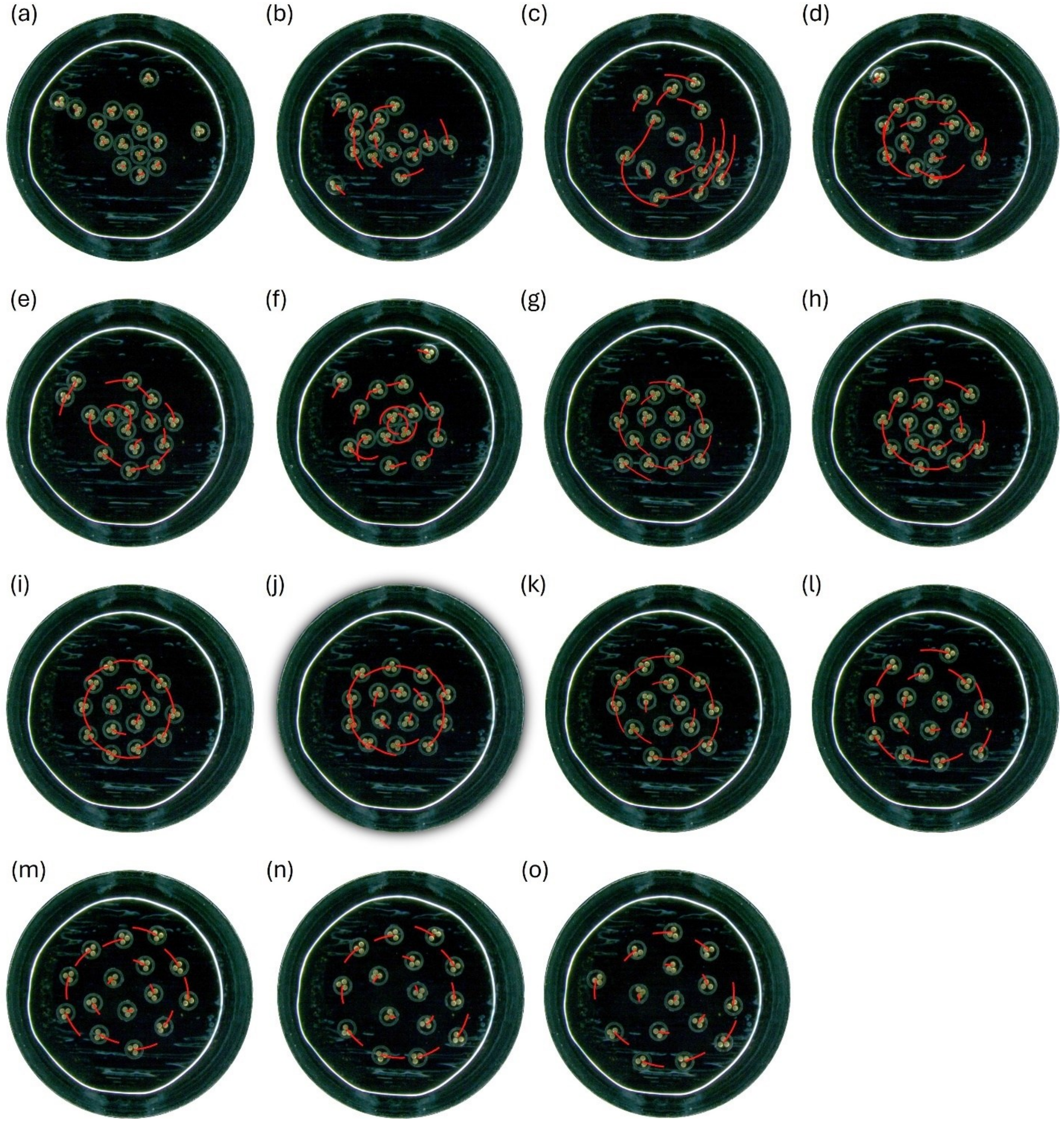

Fig. S16. Trajectory of internally driven non-concentric ring structures. (a) 1 Hz. **(b)** 5 Hz. **(c)** 10 Hz. **(d)** 15 Hz. **(e)** 20 Hz. **(f)** 25 Hz. **(g)** 30 Hz. **(h)** 35 Hz. **(i)** 40 Hz. **(j)** 50 Hz. **(k)** 60 Hz. **(l)** 70 Hz. **(m)** 80 Hz. **(n)** 90 Hz. **(o)** 100 Hz.

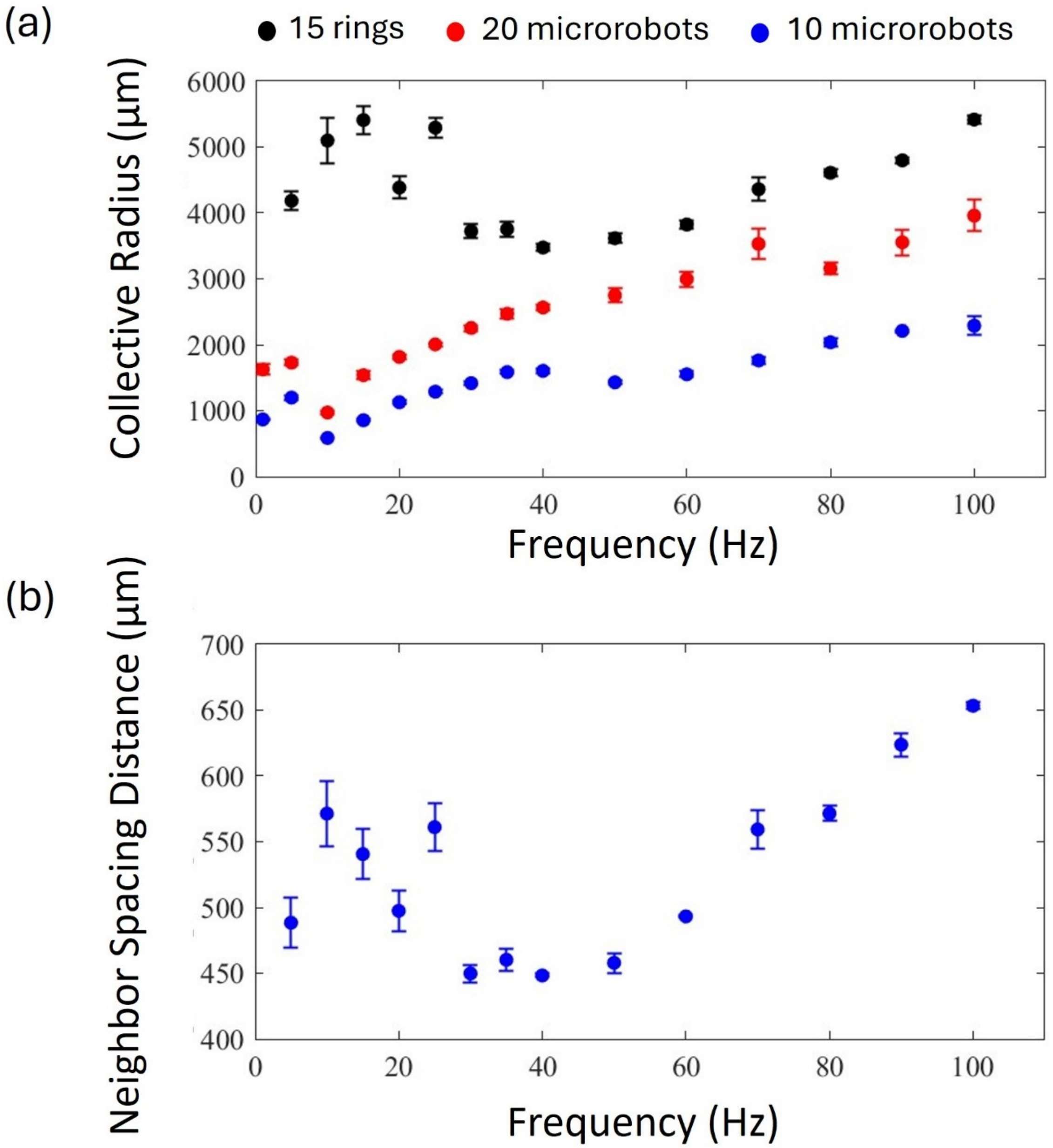

Fig. S17. Characterization of internally driven non-concentric ring structures. (a) Collective radius of the non-concentric ring structures (black), 10 microrobots (blue), and 20 microrobots (red). **(b)** Neighbor spacing distance between Voronoi neighbors.

Characterization of object absorption and expulsion with a 1000-microrobot collective

In the following experiments, we study how a ~1000 microrobot collective can control the motion and position of multiple passive objects. These experiments were run in a 40 mm by 20 mm rectangular arena to maximize the area occupied by 1000 microrobots while considering the limits of the camera view and the magnetic field workspace. When there are no passive particles in the surrounding environment, the microrobots form a rotating vortex at low frequencies (<15 Hz) and many rotating clusters at higher frequencies (>15 Hz) (Fig. S18). The Shannon entropy (H_{NDist}) quantifies how 'disorganized' the passive particles become as the field frequency increases; for the experiments with 20 objects (Figs. S19-S21), H_{NDist} increases with frequency until about Ω = 30 Hz and then levels out. The microrobot collective expands to the full size of the arena and becomes completely disordered at this range; this enables the passive particles to be distributed across the entire area. H_{NDist} is defined as the Shannon entropy of the distribution of distances between all neighbor pairs. The distribution of neighbors was calculated for all time frames and using Voronoi tessellation and distance between pairs of passive particles. Two passive particles were considered neighbors for the Shannon entropy calculation if they were Voronoi neighbors and the distance between them was less than 6.5mm; this was approximately a third of the arena width and was to ensure that particles on opposite sides of the arena that were Voronoi neighbors were not considered neighbors, since the effects they have on each other would be minimal.

$$H_{NDist} = -\sum_{i} p_i \log_2 p_i \tag{15}$$

Here, p_i is the probability of a neighbor distance value in the ith bin. At low frequencies, the passive particles travel along the edge of the arena which causes them to have a slightly organized distribution of pairwise distances with a peak around 7-8 mm and a spread towards higher and lower values; however, when the particles spread throughout the arena, they decrease the peak distance and instead have a longer spread toward the higher side. The particle-boundary distance distributions for the 20 objects are more intuitive; here, the distance is measured between each passive particle and the nearest arena edge. As the field frequency increases, the distribution flattens out to resemble a uniform distribution which means the particles are spread throughout the arena and have many particles in the center and outer regions. The histograms for pairwise particle distances for each of the tested field frequencies show the gradual transition of the shape of the distributions as the field frequency increases. The results for when there are 40 objects are shown in Figs. S22-S25. The histograms and Shannon entropy plot for the 40 objects do not have a distinct transition in the level of order of the objects from low to high frequencies. The higher number of objects means more of them will be likely to interact with the arena's boundary and once an object has touched a boundary it is more likely to stick even when the microrobots are driven at high frequencies.

The experiments presented in Fig. S26 demonstrate that the microrobots can freeze the positions of the passive particles by switching to a collectively static state. The objects have organized at the periphery because the collective was rotating under a field frequency of Ω = 5 Hz; however, once the axial field frequencies were switched to Ω_x = 5 Hz and Ω_y = 10 Hz, the objects ceased to orbit the microrobot collective and instead moved towards each other. The microrobots spin continuously about their axis when $\Omega_y = \Omega_x$ because the instantaneous magnetic field vector follows a circular trajectory which causes each constituent to align its magnetic dipole with that vector; this enables the circular flows that lead the collective to rotate and push the passive particles to the boundary at low field frequencies. When the relationship between the axial frequencies is

not one-to-one, however, the magnetic field vector traces out more complex trajectories, which causes microrobots to not spin continuously about their axis. When the axial frequencies follow the relationship $\Omega_y = 2\Omega_x$, the magnetic field vector follows a parabolic trajectory which causes the microrobots to oscillate between clockwise and counterclockwise spinning, which cancels out rotational fluid flows and enables the collective to remain static with respect to a global reference frame while its constituents continuously change their orientation. The absence of the flows means the objects also cease to move much and instead are more susceptible to the capillary interactions with other objects and the arena boundary. Since the objects have more open space to move towards each other when they are at the perimeter of the collective at low frequencies, they can move towards each other more easily because of their capillary interactions, and cluster at a specific site of the arena, as shown in Fig. S26. When the rotating field frequency is higher, Ω = 30 Hz, the passive particles mix well with the microrobots and are less prone to form large clusters after the axial frequencies are switched to Ω_x = 30 Hz and Ω_y = 60 Hz; the high number of microrobots between objects prohibit them from easily moving because of pairwise capillary interactions.

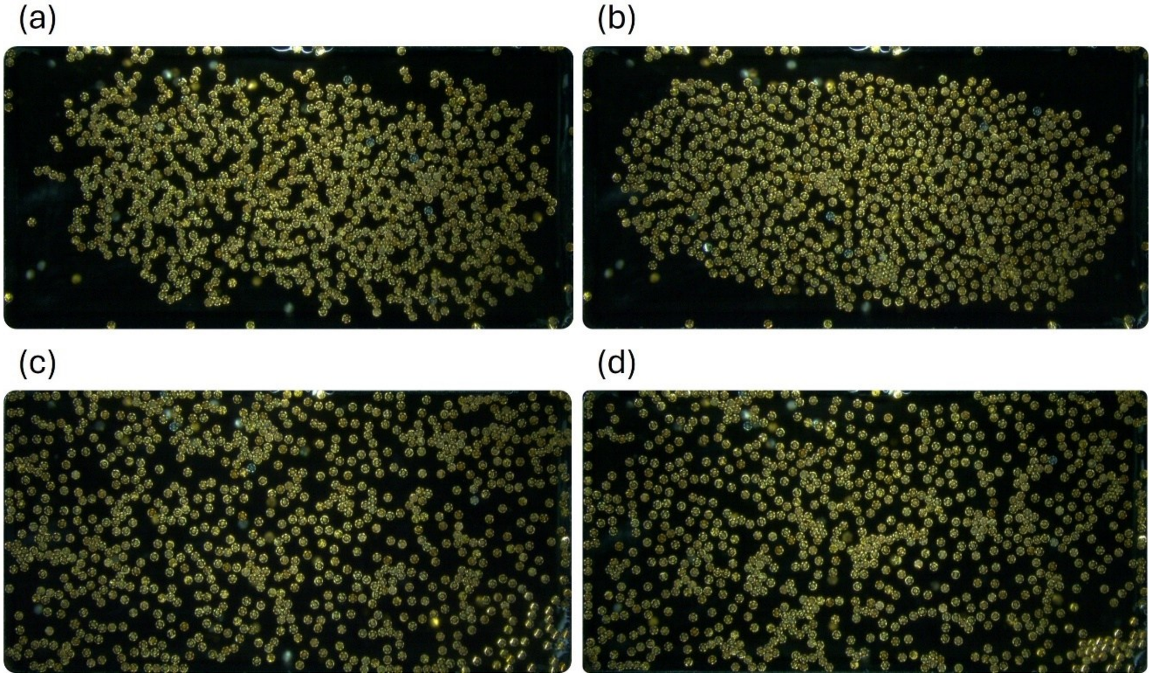

Fig. S18. 1000-microrobot collective driven at various frequencies. (a) 1 Hz. **(b)** 5 Hz. **(c)** 30 Hz. **(d)** 50 Hz.

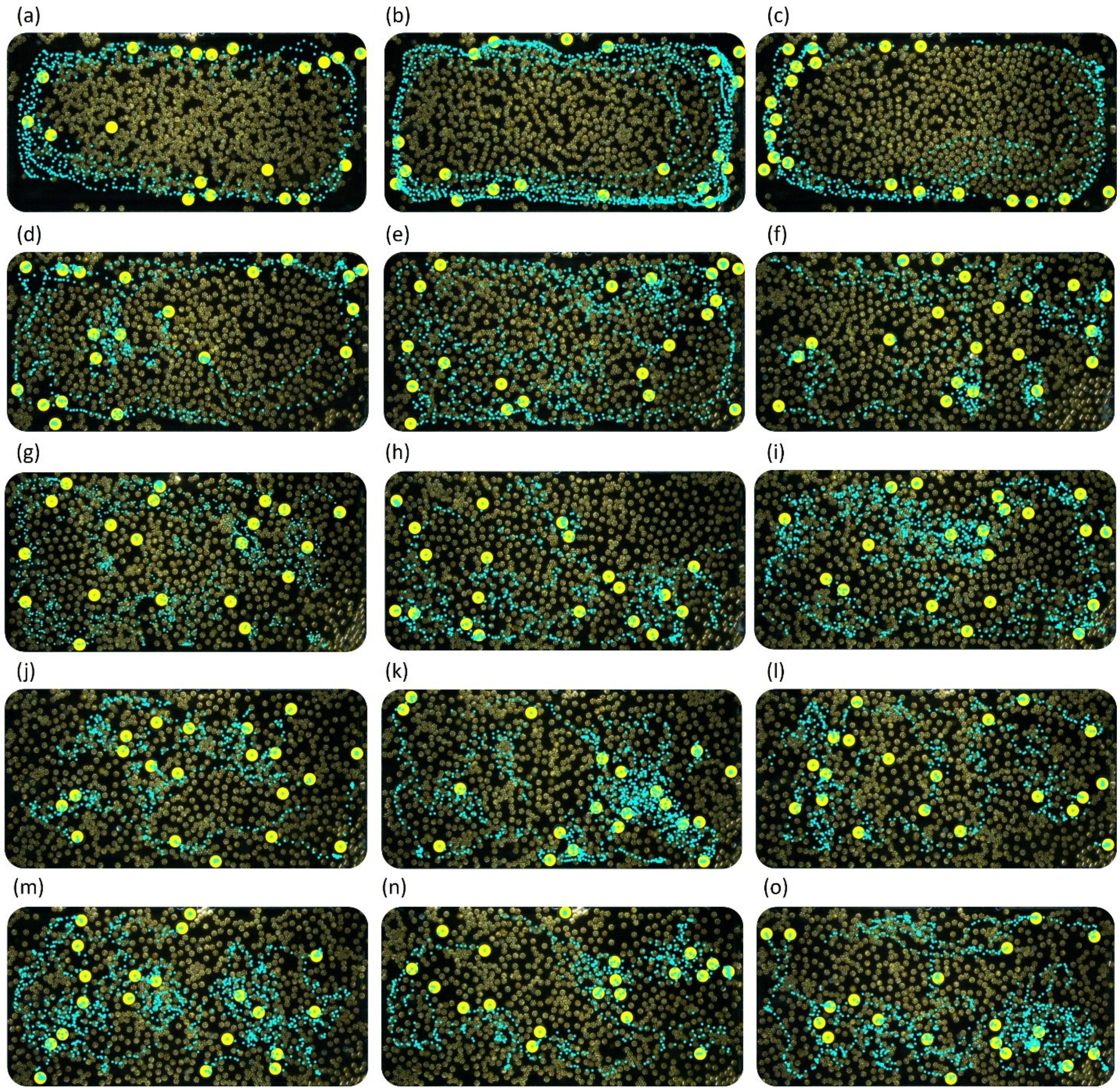

Fig. S19. Trajectories of 20 passive particles. Passive particles tend to spread more across the arena as the field frequency increases. **(a)** 1 Hz. **(b)** 5 Hz. **(c)** 10 Hz. **(d)** 15 Hz. **(e)** 20 Hz. **(f)** 25 Hz. **(g)** 30 Hz. **(h)** 35 Hz. **(i)** 40 Hz. **(j)** 45 Hz. **(k)** 50 Hz. **(l)** 55 Hz. **(m)** 60 Hz. **(n)** 65 Hz. **(o)** 70 Hz.

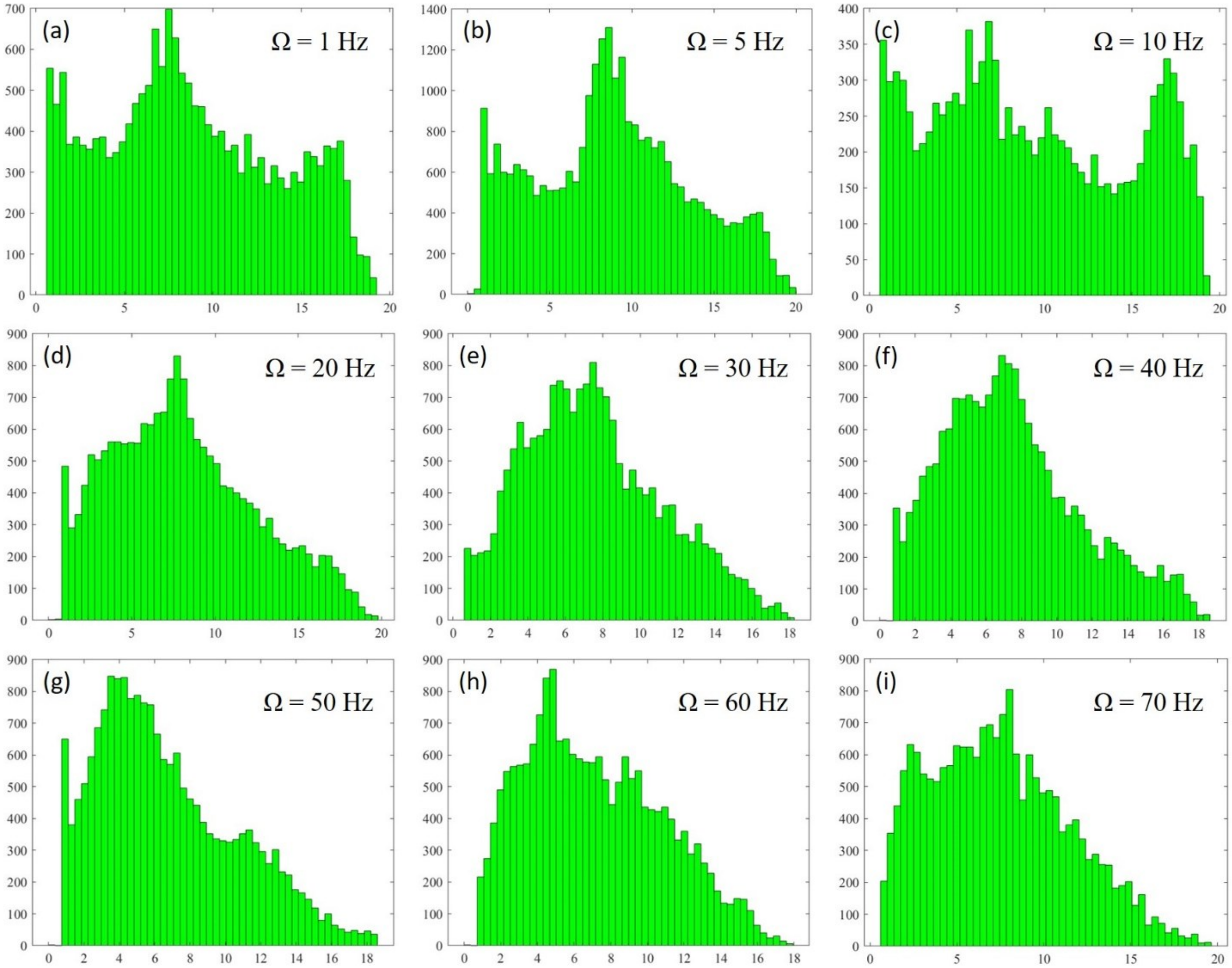

Fig. S20. Histogram of pairwise distances between 20 passive particles. Pairwise distances (in mm) were measured for ~50 time steps per frequency test. **(a)** 1 Hz. **(b)** 5 Hz. **(c)** 10 Hz. **(d)** 20 Hz. **(e)** 30 Hz. **(f)** 40 Hz. **(g)** 50 Hz. **(h)** 60 Hz. **(i)** 70 Hz.

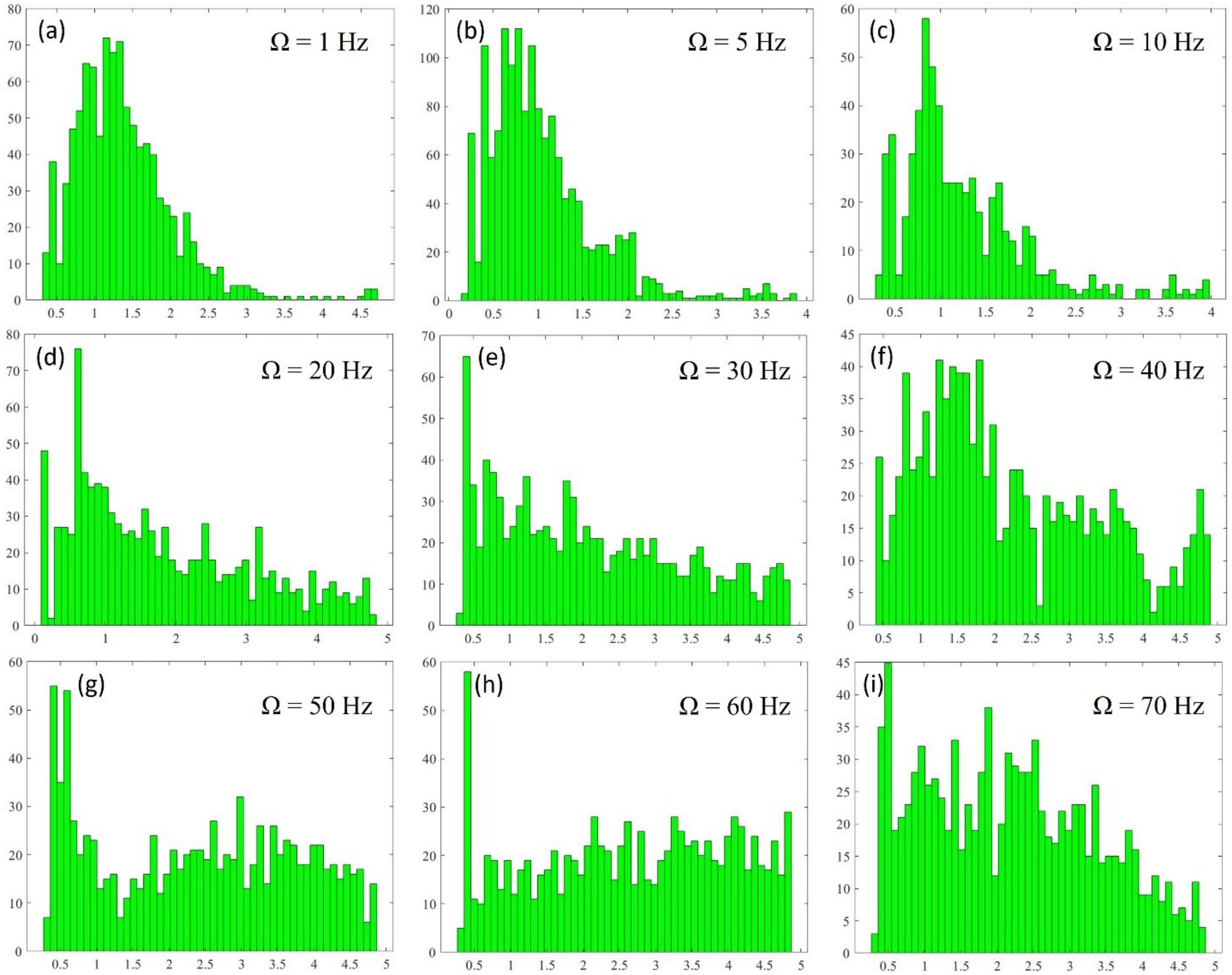

Fig. S21. Histogram of distances to arena edge for 20 passive particles. Distances between each passive particle and the nearest arena edge (in mm) were measured for ~50 time steps in each frequency test. **(a)** 1 Hz. **(b)** 5 Hz. **(c)** 10 Hz. **(d)** 20 Hz. **(e)** 30 Hz. **(f)** 40 Hz. **(g)** 50 Hz. **(h)** 60 Hz. **(i)** 70 Hz.

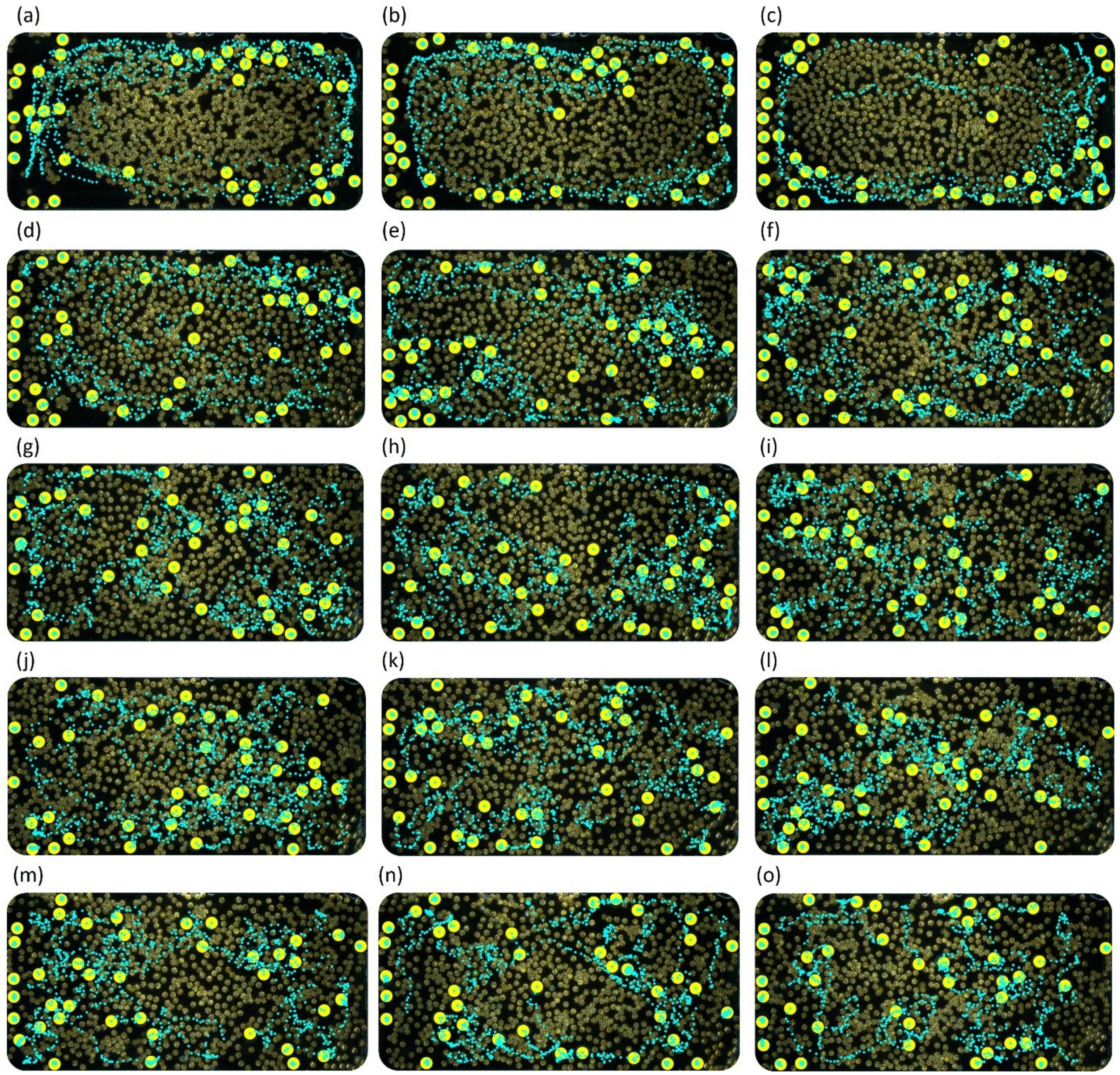

Fig. S22. Trajectories of 40 passive particles. Passive particles tend to spread more across the arena as the field frequency increases. **(a)** 1 Hz. **(b)** 5 Hz. **(c)** 10 Hz. **(d)** 15 Hz. **(e)** 20 Hz. **(f)** 25 Hz. **(g)** 30 Hz. **(h)** 35 Hz. **(i)** 40 Hz. **(j)** 45 Hz. **(k)** 50 Hz. **(l)** 55 Hz. **(m)** 60 Hz. **(n)** 65 Hz. **(o)** 70 Hz.

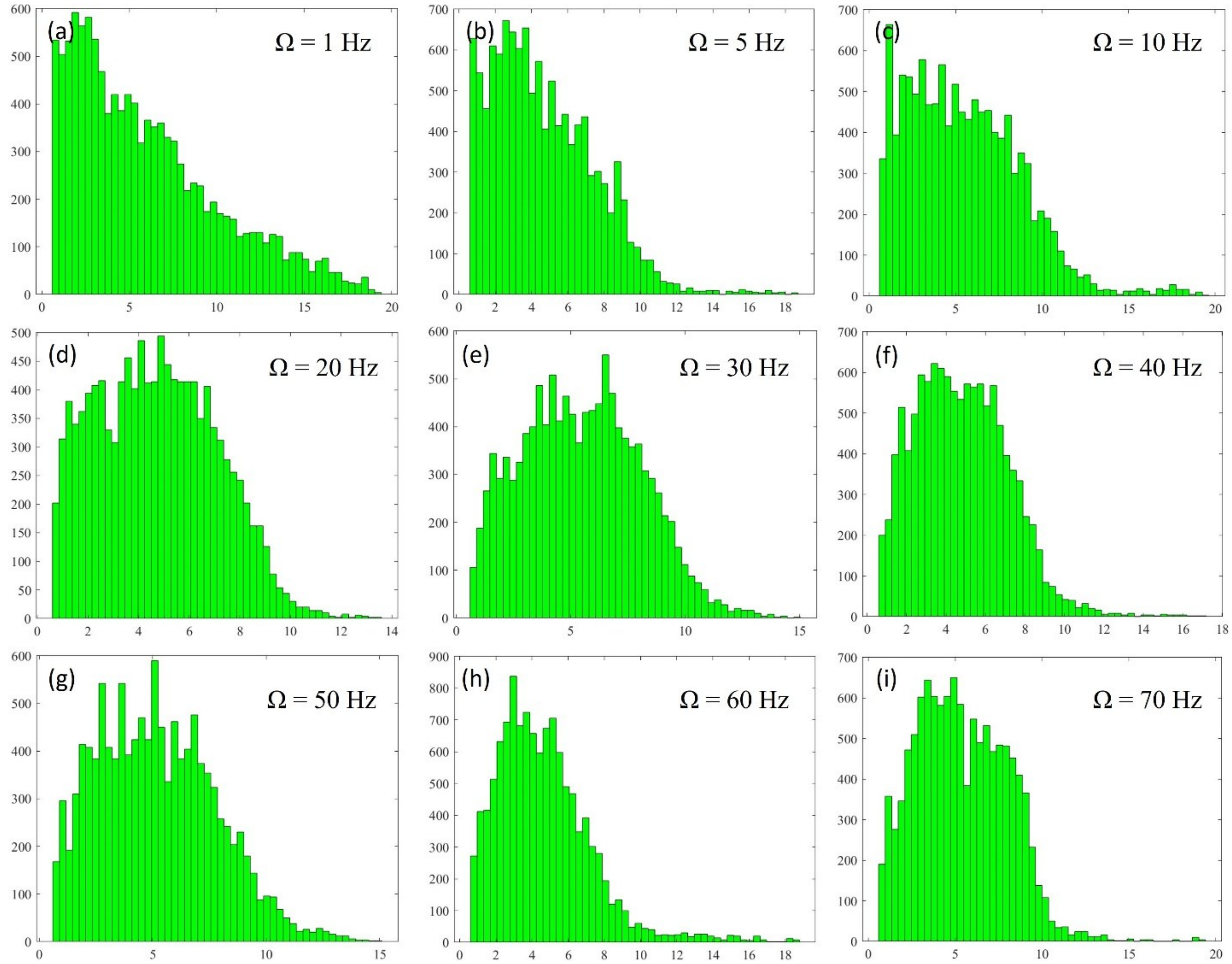

Fig. S23. Histogram of pairwise distances between 40 passive particles. Pairwise distances (in mm) were measured for ~50 time steps per frequency test. **(a)** 1 Hz. **(b)** 5 Hz. **(c)** 10 Hz. **(d)** 20 Hz. **(e)** 30 Hz. **(f)** 40 Hz. **(g)** 50 Hz. **(h)** 60 Hz. **(i)** 70 Hz.

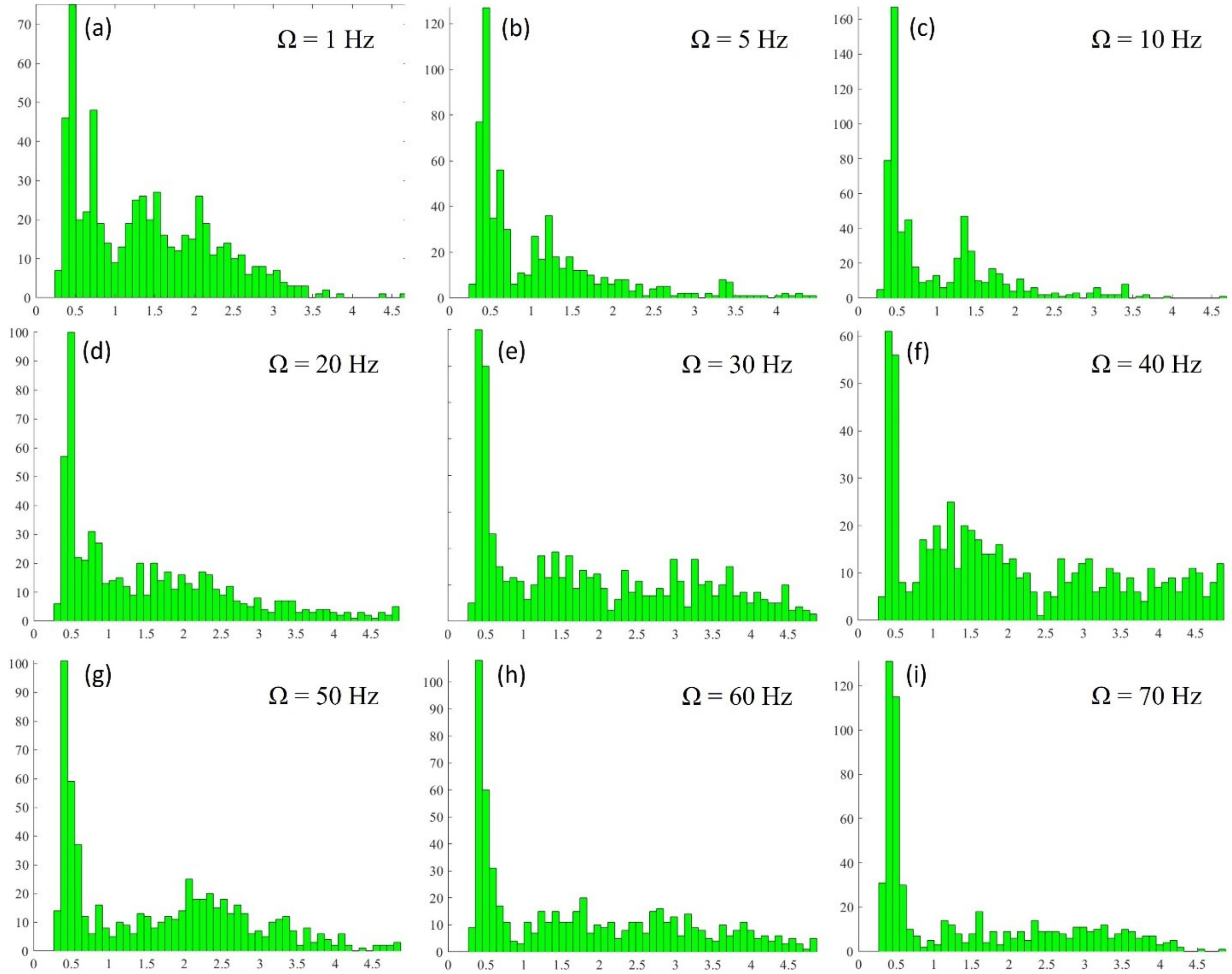

Fig. S24. Histogram of distances to arena edge for 40 passive particles. Distances between each passive particle and the nearest arena edge (in mm) were measured for ~50 time steps in each frequency test. **(a)** 1 Hz. **(b)** 5 Hz. **(c)** 10 Hz. **(d)** 20 Hz. **(e)** 30 Hz. **(f)** 40 Hz. **(g)** 50 Hz. **(h)** 60 Hz. **(i)** 70 Hz.

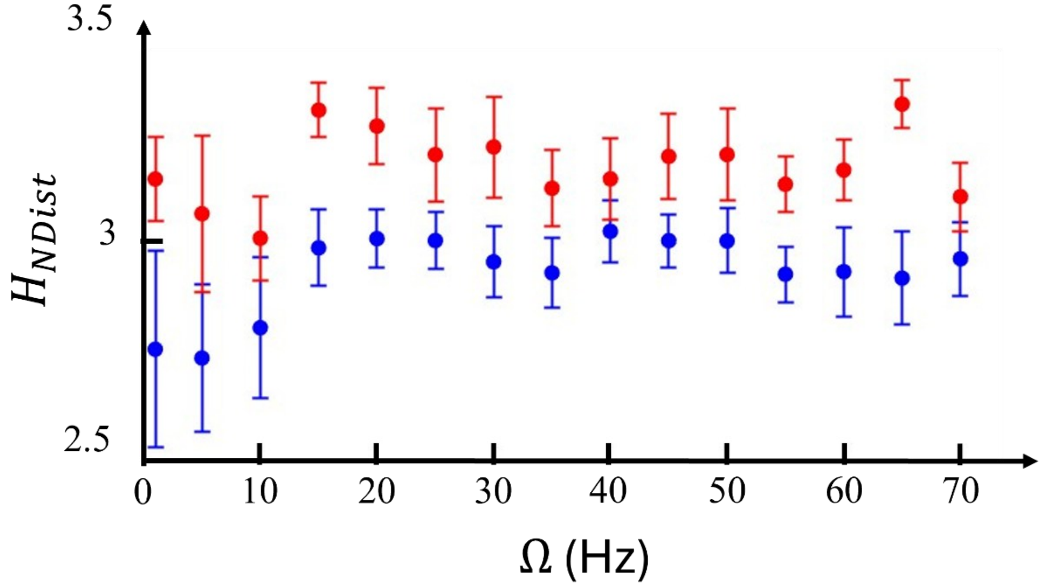

Fig. S25. Shannon entropy of circular objects manipulated by 1000 microrobots. Shannon entropy (H_{NDist}) for 20 circular objects (blue) and 40 circular objects (red).

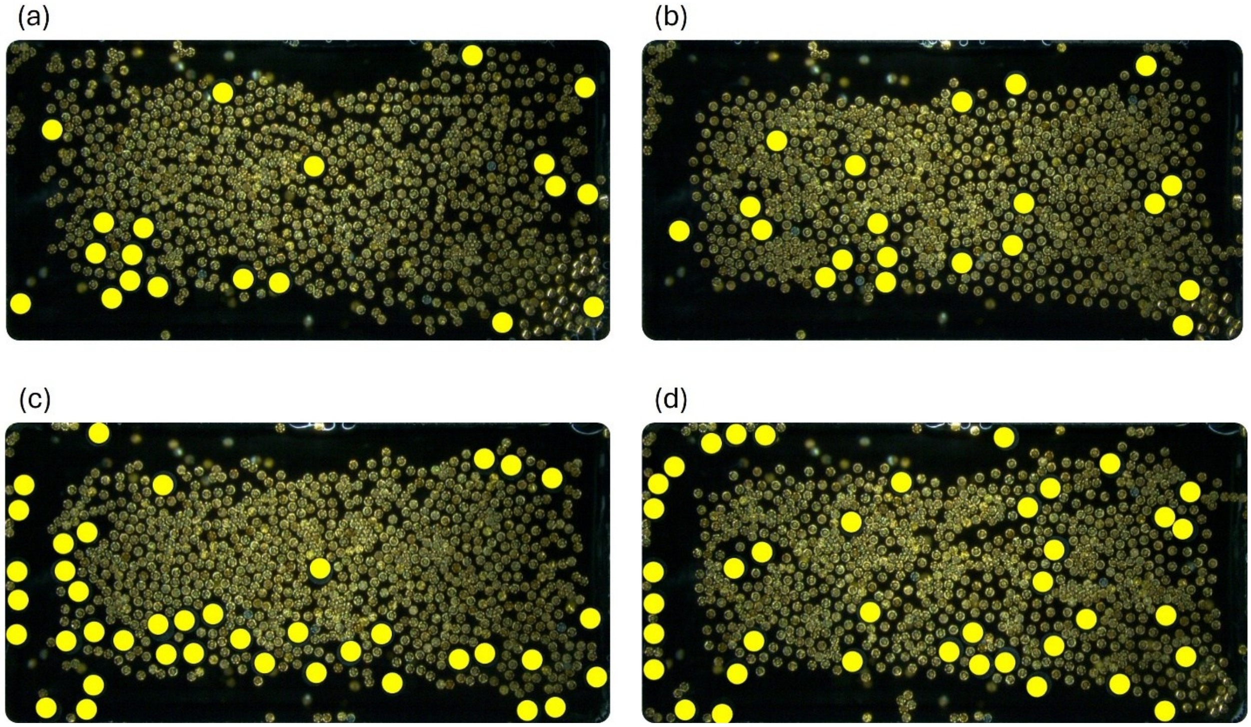

Fig. S26. Object positions while manipulated by a static 1000-microrobot collective. (a-b) 20 circular objects. **(a)** $\Omega_x = 10$ Hz, $\Omega_y = 20$ Hz. **(b)** $\Omega_x = 30$ Hz, $\Omega_y = 60$ Hz. (c-d) 40 circular objects. **(c)** $\Omega_x = 10$ Hz, $\Omega_y = 20$ Hz. **(d)** $\Omega_x = 30$ Hz, $\Omega_y = 60$ Hz.

Discussion on collective crawling about passive objects

The geometry of the object plays a big role in whether the collective crawls as a single cluster (main text Fig. 4B) or it splits up into several groups that each crawl along the perimeter of the object (main text Fig. 4D). The gear shown in main text Fig. 4D has concave features that disrupt the azimuthal flow fields and causes a cluster to remain within a local concave cavity of the object while the rest of the collective moves along the perimeter. Since the collective is much larger than the total area of the gear's concave features, a large portion of the microrobots can continue crawling along the edge of the object; however, as it moves along the convex and concave portions, it purges or gains several microrobots in the vicinity. The rectangular object, shown in main text Fig. 4E, also disrupts the azimuthal flow fields and can cause the collective to leave microrobots behind on opposite sides of the object. The rectangular object rotates much slower at the mid-range frequencies because the microrobots do not surround its entire perimeter; this leads to higher translation than rotation. The separation is not as pronounced in these cases since it always remains in one or two groups; however, in the gear and rod cases, the aggregated cluster moves about the object's perimeter continuously and the larger of the two groups tends to move faster and it gains and purges microrobots as it surpasses the other group.

For the experiments on aggregates of different sizes, the highest angular velocity is achieved for aggregates of 20 objects ($N_{obj} = 20$) at $\Omega = 40$ Hz (main text Fig. 4G), because the smaller size of the aggregate mass means the microrobot collective needs to generate less fluidic torque to rotate the object at high speeds. Note that the angular velocity decreases a bit as Ω increases when $N_{obj} = 20$; this is due to the higher hydrodynamic repulsion, which enables microrobots to spread out, and as they move farther away from the edge of the aggregate, the fluidic torque generated by the outer microrobots does not influence the aggregate as much because they are farther away. There is significantly less rotation at higher N_{obj} (main text Figs. 4H-I), and when $N_{obj} = 100$, the aggregate's angular velocity remains low across all frequencies because the size of the aggregate object causes higher amounts of fluidic drag as it rotates over the fluid surface. The shape of these objects also plays an important role in the microrobot collective's morphology at the lower frequencies. In the experiments shown in main text Figs. 4A-E, the objects have outer perimeters that are either completely convex boundaries or have large convex and concave regions; both conditions enable the collective to form an aggregated mass that 'crawls' about the perimeter of the objects. In the experiments in main text Figs. 4G-I, the aggregate is made up of small circular objects that create very small convex and concave regions, and the concave regions partially trap the microrobots such that the collective is not able to form the more cohesive crawling mass, but instead small clusters that crawl along the perimeter of the object; the crawling state persists to higher frequencies because small microrobot clusters remain trapped within these concave regions at the higher frequencies. This is reflected through the difference in angular velocities of the circular objects and the aggregate masses (main text Fig. 4K), where the aggregate masses have similar areas to the circular objects but exhibit slightly lower angular velocities because their corrugations trap clusters of microrobots.

Supplementary Movies

Movie S1.
Two concentric rings with microrobots only in the annulus region. Counter rotation of two concentric rings driven by microrobots in the annulus region between the two rings.

Movie S2.
Two concentric rings with 10 microrobots in the center region. Co- and Counter-rotation of two concentric rings driven by 10 microrobots in the center region and 10-90 microrobots in the annulus region between the two rings.

Movie S3.
Two concentric rings with 20 microrobots in the center region. Co- and Counter-rotation of two concentric rings driven by 20 microrobots in the center region and 10-90 microrobots in the annulus region between the two rings.

Movie S4.
Two concentric rings with 30 microrobots in the center region. Co- and Counter-rotation of two concentric rings driven by 20 microrobots in the center region and 10-90 microrobots in the annulus region between the two rings.

Movie S5.
Three concentric ring structures with microrobots in the center and annulus regions. Co- and counter-rotation of three concentric rings driven by 15 microrobots in the center region, 30 microrobots in the inner annulus region, and 60 microrobots in the outer annulus region.

Movie S6.
Simulations of rotating concentric rings at 70 Hz. Simulations of co- and counter-rotating rings driven by microrobots in the center and annulus regions.

Movie S7.
Internally driven gear-like structures. Microrobots drive the rotation of fixed and free-floating gear-like structures.

Movie S8.
Internally driven ring structures with fluid interactions. Dynamic self-assembly of 15 non-concentric rings each internally driven by three microrobots.

Movie S9.
1000 microrobot collective in rotation and static mode. 1000 microrobots exhibit rotating and static collective behaviors at various frequencies.

Movie S10.
1000 microrobot collective absorbing and expelling 20 objects. 1000 microrobots absorb and expel 20 circular objects so they rotate around the perimeter of the collective or are dispersed throughout the collective's coverage area.

Movie S11.
1000 microrobot collective absorbing and expelling 40 objects. 1000 microrobots absorb and expel 40 circular objects so they rotate around the perimeter of the collective or are dispersed throughout the collective's coverage area.

Movie S12.
Crawling-like behavior and object rotation. 1000 microrobots exhibit crawling-like motion about the perimeter of various objects and rotate aggregates of objects of increasing sizes.
